# Multi‐omics profiling reveals key factors involved in Ewing sarcoma metastasis

**DOI:** 10.1002/1878-0261.13788

**Published:** 2025-01-05

**Authors:** Mariona Chicón‐Bosch, Sara Sánchez‐Serra, Marta Rosàs‐Lapeña, Nicolás Costa‐Fraga, Judit Besalú‐Velázquez, Janet Illa‐Bernadí, Silvia Mateo‐Lozano, Florencia Cidre‐Aranaz, Thomas G.P. Grünewald, Ángel Díaz‐Lagares, Roser Lopez‐Alemany, Òscar M. Tirado

**Affiliations:** ^1^ Sarcoma Research Group Institut d'Investigació Biomèdica de Bellvitge (IDIBELL), Oncobell, L'Hospitalet de Llobregat Barcelona Spain; ^2^ Universitat de Barcelona (UB) Barcelona Spain; ^3^ Epigenomics Unit, Cancer Epigenomics, Translational Medical Oncology (ONCOMET), Health Research Institute of Santiago (IDIS) University Clinical Hospital of Santiago (CHUS/SERGAS) Santiago de Compostela Spain; ^4^ Galician Precision Oncology Research Group (ONCOGAL), Medicine and Dentistry School Universidade de Santiago de Compostela (USC) Spain; ^5^ Universidad de Santiago de Compostela (USC) Spain; ^6^ Department of Clinical Analysis University Hospital Complex of Santiago de Compostela (CHUS) Spain; ^7^ Developmental Tumor Biology Laboratory Institut de Recerca Sant Joan de Déu, Hospital Sant Joan de Déu Barcelona Spain; ^8^ Pediatric Cancer Center Barcelona Hospital Sant Joan de Déu Barcelona Spain; ^9^ Division of Translational Paediatric Sarcoma Research German Cancer Research Center (DKFZ), German Cancer Consortium (DKTK) Heidelberg Germany; ^10^ Hopp‐Children's Cancer Center (KiTZ) Heidelberg Germany; ^11^ National Center for Tumor Diseases (NCT) NCT Heidelberg, a partnership between DKFZ and Heidelberg University Hospital Germany; ^12^ Institute of Pathology Heidelberg University Hospital Heidelberg Germany; ^13^ CIBERONC Carlos III Institute of Health (ISCIII) Madrid Spain; ^14^ Institut Català d'Oncologia (ICO) L'Hospitalet de Llobregat Barcelona Spain

**Keywords:** CREB1, Ewing sarcoma, FGD4, LOXHD1, metastasis, methylomics, mouse model, multi‐omics, proteomics, transcriptomics

## Abstract

Ewing sarcoma (EWS) is the second most common bone tumor affecting children and young adults, with dismal outcomes for patients with metastasis at diagnosis. Mechanisms leading to metastasis remain poorly understood. To deepen our knowledge on EWS progression, we have profiled tumors and metastases from a spontaneous metastasis mouse model using a multi‐omics approach. Combining transcriptomics, proteomics, and methylomics analyses, we identified signaling cascades and candidate genes enriched in metastases that could be modulating aggressiveness in EWS. Phenotypical validation of two of these candidates, cyclic AMP‐responsive element‐binding protein 1 (CREB1) and lipoxygenase homology domain‐containing protein 1 (LOXHD1), showed an association with migration and clonogenic abilities. Moreover, previously described CREB1 downstream targets were present amongst the metastatic‐enriched results. Combining the different omics datasets, we identified FYVE, RhoGEF, and PH domain‐containing protein 4 (FGD4) as a CREB1 target interconnecting the different EWS biological layers (RNA, protein and methylation status) and whose high expression is associated with worse clinical outcome. Further studies will provide insight into EWS metastasis mechanisms and ultimately improve survival rates for EWS patients.

## Graphical abstract

Using a mouse model that recapitulates the spread of tumor cells within the body, we have profiled paired tumors and metastasis samples using a multiomics approach combining different layers of biological information. We identified key genes that drive metastasis in Ewing sarcoma.

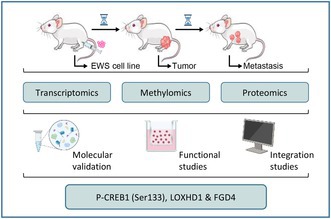

**Abbreviations**
BP: biological process
CBP: CREB‐binding protein
CpGIs: CpG islands
CRE: cAMP response element
DEG: differentially expressed genes
DMCpGs: differentially methylated CpGs
EWS: Ewing sarcoma
FC: fold change
FDR: false discovery rate
GO: gene ontology
GSEA: gene set enrichment analysis
IPA: ingenuity pathway analysis
KO: knock out
PCA: principal component analysis
SEM: standard deviation of the mean

## 1 Introduction

Sarcomas comprise a diverse group of cancers that arise in bones and soft tissues, with a wide spectrum of molecular and histological characteristics [1]. Identifying the cell of origin or the molecular mechanism behind these malignancies is proven difficult, resulting in a struggle to find effective treatment strategies for sarcoma patients [2]. Ewing sarcoma (EWS) is the second most common primary bone tumor in children, adolescents and young adults [3]. It is an aggressive cancer associated with poor survival rates, especially for those patients that present with metastasis at diagnosis (25% of EWS), with a 5‐year overall survival rate under 30% [4]. Despite improvements in deciphering the mechanisms behind EWS tumorigenesis, such as the identification of the EWSR1::ETS translocation [5], survival rates for patients with metastatic disease have remained stagnant. For this reason, many groups in the EWS field are investigating EWS with the aim of identifying better treatment strategies that would improve survival rates, with a special focus on metastatic patients.

With the introduction of omics techniques to study cancer biology, many groups in oncology are studying cancer from a wider perspective rather than focusing on specific signaling pathways [6]. Through this approach, the different mechanisms involved in the biology of cancer can be identified, as well as the pathways that are being activated and which candidates lead to phenotypic changes in cells. EWS has long been known as a silent cancer, with only a few genetic alterations besides the oncogenic translocation [7, 8] and a recent connection between the EWSR1::FLI1 fusion and GGAA‐containing microsatellite elements that regulate downstream signaling [9]. However, many groups have extended their studies also on other aspects of EWS's biology, such as transcriptomics including single‐cell RNA‐seq [10, 11, 12], proteomics [13, 14, 15, 16, 17], and epigenomics [18, 19, 20]. Using these approaches, EWS molecular signaling is being evaluated, identifying specific mechanisms that can be further interrogated *in vitro* and *in vivo*. Recently, two studies using multi‐omics approaches in EWS have been published, which investigated the profile of EWS cell lines combining a variety of high‐throughput omics techniques [21, 22]. Altogether, these studies help us deepen our understanding on how EWS cells activate, progress and metastasize, and how best we could target and eliminate them.

Here, for the first time, we employ a multi‐omics approach to study the process of metastasis in EWS. Based on a spontaneous metastasis mouse model developed in our laboratory [23], we compared EWS primary tumors and metastases from our *in vivo* model to identify the changes that occur in EWS cells throughout progression. Results on transcriptomics, proteomics, and methylomics from these samples are shown and discussed, providing some insight into what mechanisms might be fundamental for EWS disease progression. We have identified several candidates and mechanisms that could be essential for the development of metastasis in EWS, moving the field forward into understanding this challenging disease. Ultimately, we hope they take us closer in developing efficient targeted therapies that will lead to improved survival rates in EWS.

## 2 Materials and methods

### 2.1 Ethics statement

Animals were cared for according to the Institutional Guidelines for the Care and Use of Laboratory Animals. Ethics approval was provided by Catalan Government Animal Care Committee (permit no. 9745, according to the RD53/2013). IDIBELL animal facility abides by the Association for Assessment and Accreditation of Laboratory Animal Care (AAALAC) regulations.

### 2.2 Cell culture

Ewing sarcoma cell lines: A673 (RRID: CVCL_0080) and TC‐252 (RRID: CVCL_S866; gifts from Dr. Heinrich Kovar), A4573 (RRID: CVCL_6245; gift from Dr. Santiago Ramón y Cajal). Cell lines were cultured in RPMI 1640‐GlutaMAX (Life Technologies, Carlsbad, CA, USA) supplemented with 10% (v/v) fetal bovine serum (FBS; Life Technologies) and 1% (v/v) penicillin–streptomycin (P/S; Life Technologies). All cell lines were incubated at 37 °C in a humidified atmosphere of 5% CO_2_ in air and checked regularly for mycoplasma contamination using DNA staining with Hoechst 33342 dye (Life Technologies). Exponentially growing cells were used for all experiments. Authenticity of the cell lines was routinely confirmed by STR profiling analysis.

A673 transfected with luciferase expressing construct (A673Luc; gift from Dr. Ibane Abasolo, VHIR, Barcelona), was cultured in antibiotic selection media (puromycin; Sigma‐Aldrich, Saint Louis, MO, USA). These cells were used for the *in vivo* experiments as they could be detected and imaged using luciferase activity through *In Vivo* Imaging System (IVIS®; PerkinElmer, Shelton, CT, USA). TC‐252 cells for *in vivo* experiments were cultured following the same protocol.

Metastasis‐derived primary cultures from A673Luc and TC‐252 mice lung metastasis were obtained (see below). Lung metastatic tissue was placed in cell culture plates with RPMI +1% P/S, ground with a scalpel and centrifuged at 600 ***g*** for 5 min. Tissue pellet was resuspended in RMPI +1% P/S and collagenase II 0.1% and incubated for 2 h at 37 °C shaking every 20 min. Same volume of RPMI + 20% FBS + 1% P/S was added and filtered using a 70‐μm Nylon filter (BD Falcon, Schaffhausen, Switzerland) and any tissue remaining on the filter was scraped off. Tissue was centrifuged at 1400 ***g*** for 10 min, resuspended with RPMI + 20% FBS + 1% P/S and placed in a cell culture plate at a confluency of 1‐2 × 10^6^ cells/p60 plate. Once cells reached 80% confluency, they were cultured as standard cell lines (described before). Primary cultures obtained from A673Luc lung metastases named as Met1‐2‐3A6; those obtained from TC‐252 lung metastases named as Met2‐3‐4‐5TC.

### 2.3 Spontaneous metastasis mouse model

Orthotopic model for metastatic detection in mice was performed as described [23] using 6‐week‐old female, athymic nude mice Hsd:Athymic Mice‐*Foxn1nu* from Envigo (Indianapolis, IN, USA). Luciferase‐labeled A673 EWS cells (A673Luc; 1 × 10^6^ cells) or TC‐252 EWS cells (not luciferase‐labeled; 2.5 × 10^6^ cells) were injected into the gastrocnemious muscle of mice. Tumor size was monitored using calipers. Once the tumor reached approximately 800 mm^3^, around day 15–20, the muscles containing the tumor were surgically resected (evaluation by size/weight and also IVIS lecture for A673Luc). After surgery, mice could survive for a period long enough to enable the development of distant metastases. The presence of morbidity symptoms was checked every 48 h. At day 90 after cell inoculation, lung metastases were detected by *in vivo* lecture of luminescence (IVIS) of the whole mouse (for A673Luc) or *ex vivo* once lungs resected. Mice were euthanized, and lungs were extracted and analyzed for metastasis. Some of the mice developed local relapses in areas adjacent to the surgery site and were euthanized before the experiment end point, but also included in the analysis. Animals were cared according to the Institutional Guidelines for the Care and Use of Laboratory Animals. All mice were kept in standard polycarbonate cages, 5 mice/cage, and fed sterile food and water available *ad libitum*. The mouse laboratory was pathogen‐free condition, at a controlled temperature (21–23 °C) and humidity (50–60%) and kept on a 12 h:12 h light:dark cycle. Ethics approval was provided by a locally appointed ethics committee from IDIBELL, Barcelona, Spain. IDIBELL animal facility abides by the Association for Assessment and Accreditation of Laboratory Animal Care (AAALAC) regulations.

### 2.4 Transcriptomic array and analysis (Clariom™ D array)

Total RNA (2 μg) was extracted from samples (*n* = 20) using the NucleoSpin RNA II (mRNA) from Macherey‐Nagel (Düren, Germany), following manufacturer's instructions including a DNase treatment step. Quantification and first quality control were performed on a NanoDrop ND‐1000 (Thermo Fisher Scientific, Waltham, MA, USA). Quality control assessment with Agilent 2100 Bioanalyzer (Agilent Technologies, Santa Clara, CA, USA) was performed before microarray experiments. Only samples with RNA Integrity Number (RIN) above 7 were processed. Gene expression microarray was performed with Clariom D Array (Themo Fisher Scientific) at *Servei d'Anàlisi de Microarrays* (*MARGenomics, Institut Hospital del Mar d'Investigacions Mèdiques* (IMIM), Barcelona, Spain). Amplification, labeling and hybridization were performed according to the GeneChip WT PLUS Reagent kit and the samples were hybridized to Clariom D Human (Thermo Fisher Scientific) in a GeneChip Hybridization Oven 640. Washing and scanning were performed using the Expression Wash, Stain, and Scan Kit (Affymetrix Inc., Thermo Fisher Scientific).

Gene expression (Clariom D) array data was analyzed at the Sarcoma Research Group. After quality control of raw data (aroma.affymetrix package [24]), samples were background corrected and normalized using the robust multi‐chip average (oligo package [25]) method. In order to detect DEG between sample groups, the Linear Models for Microarray (limma [26]) package was used. Correction for multiple comparisons was performed using false discovery rate (FDR). Transcripts with adjusted *P*‐value <0.05 (or *P*‐value <0.05 when stated) and absolute FC above 1.5 (absolute log_2_FC > 0.58) were selected as significant. Figures were performed using ggplot2 package [27] and rcolorbrewer package [28]. All analyses were performed in R (v3.5.2, http://www.R‐project.org/).

### 2.5 Genome‐wide DNA methylation analysis by EPIC arrays

DNA was extracted following the QIAamp DNA Mini Kit (Qiagen, Venlo, The Netherlands). Briefly, 25 mg of tumor/metastatic tissues (*n* = 3/5) from the EWS spontaneous metastasis mouse model were used. Treatments with protease K (Qiagen) and RNase A (Qiagen) were used to avoid contamination of proteins and RNAs. Methods were performed as described previously [29]. Briefly, total genomic DNA (500 ng) was converted by sodium bisulfite using the EZ DNA Methylation kit (Zymo Research, Irvine, CA, USA). Following the manufacturer's protocol, the bisulfite‐converted DNA was hybridized to the Infinium MethylationEPIC BeadChips (Illumina, San Diego, CA, USA), which cover over 850 000 CpG sites along the human genome [30]. Whole‐genome amplification and hybridization were performed, followed by single‐base extension and analysis on a HiScan (Illumina) to assess the cytosine methylation states. The methylation data were processed in the R statistical environment using RnBeads 2.0 [31]. Raw intensity data files (IDATs) were imported into RnBeads 2.0 for quality control and preprocessing. First, a greedycut algorithm was used to filter out low‐quality probes. Probes overlapping with SNPs and probes whose sequences mapped to multiple genomic locations (cross‐reactive) were removed. IDATs obtained in the array were normalized using the beta‐mixture quantile (BMIQ) method. The DNA methylation level was represented as the average β‐value, which was calculated as the ratio of the fluorescent signal intensity of the methylated probe to those of total (methylated and unmethylated) probes. Average β‐values were used to calculate the mean methylation difference between groups. Hierarchical linear models performed with RnBeads 2.0 were used to obtain the methylation differences between groups. *P*‐values were corrected for multiple testing (FDR) using Benjamini–Hochberg method and a threshold of *P*‐value <0.05 was selected for significance. Principal component analysis (PCA) and unsupervised hierarchical clustering heatmaps of β‐values were generated using the prcomp function and Complex Heatmap package, respectively. The enrichment analysis of biological pathways was evaluated by gene ontology (GO) using Genecodis [32].

### 2.6 DNA methylation analysis by pyrosequencing

A total of 500 ng of genomic DNA for each sample was used for the bisulfite conversion with the EZ DNA Methylation kit (Zymo Research) following manufacturer's recommendations. DNA methylation levels of CpGs were analyzed by pyrosequencing as previously described [33]. Methods were performed as described previously [34]. Briefly, primer sequences were designed with PyroMark Assay Design 2.0 (Qiagen). Standard PCR reactions were carried out with bisulfite‐converted genomic DNA. PCR products were observed in 2% agarose gels before pyrosequencing. PyroMark Q24 Vacuum Workstation was used for the immobilization and preparation of PCR products. Pyrosequencing reactions were performed using a PyroMark Gold Q24 Reagent Kit (Qiagen) following the manufacturer's instructions. Methylation values were obtained using PyroMark Q24 Software 2.0 (Qiagen). Assays were conducted including methylated and unmethylated DNA controls from Zymo Research, and water was used as no template control.

### 2.7 Cell treatments (Decitabine and 666‐15 inhibitor)

The day after seeding, cells were washed with PBS and 2.5 μm of Decitabine (5‐Aza‐2′‐deoxycytidine; Sigma‐Aldrich) or CREB1 inhibitor 666‐15 (Sigma‐Aldrich) [35] was added to the cells in complete media. DMSO was used as vehicle control for all experiments as both drugs were dissolved in it. For Decitabine, cells were collected after 72 h of treatment (cells re‐treated every 24 h) and used for gene expression analysis. For CREBi, cells were collected after 24–48 h depending on downstream experiment (protein expression or phenotypic validation).

### 2.8 Proteomic array and analysis (Phospho explorer antibody array)

Proteomic expression analyses were done using the Phospho Explorer Antibody Array (Full Moon Biosystems, TebuBio, Le Perray en Yvelines, France). Protein extraction was performed following the Antibody Array user's guide to avoid incompatibilities with the downstream hybridization and biotinylation of samples to the array. Briefly, 30 mg of paired tumor/metastasis (*n* = 3/3) from the spontaneous metastasis mouse model using A673Luc cell line were processed as described in the Antibody Array user's guide (Full Moon BioSystems) using protease/phosphatase inhibitors as suggested (Roche, Basel, Switzerland). Proteins were quantified using the Pierce BSA kit (Thermo Fisher Scientific) and concentrated using Centricon 10 k filters (Amicon Ultra‐15 centrifugal filters, Millipore, Burlington, VT, USA) to reach a 1 μg/μl protein concentration. For the downstream protocol, 50 μg of protein was used. For the protein labelling (biotinylation), blocking, coupling and detection, the Antibody Array user's guide was followed. Slides were prepared for shipping and sent to Tebu‐bio laboratories (Le Perray en Yvelines, France) where they were scanned and analyzed using their protocol (not disclosed). Raw data of proteomics analysis supplied as Tables [Link], [Link], [Link], [Link].

### 2.9 Pathway analyses and integration of data

Functional analyses for the different omics datasets were performed on the Gene Set Enrichment Analysis (GSEA) desktop software using the pre‐ranked tool [36, 37], using collections H (Hallmark), C2 (Curated gene set; KEGG), and C5 (gene ontology gene set; GO). The QIAGEN Ingenuity Pathway Analysis (IPA) was used to identify pathway regulators and networks on our transcriptomic data [38]. Overlap analyses between the different datasets were performed using the Venn diagram tool from University of Gent Bioinformatics department (https://bioinformatics.psb.ugent.be/webtools/Venn/).

### 2.10 Reverse transcription quantitative PCR (RT‐qPCR)

RNA extraction from cell pellets was performed as described in Transcriptomic arrays section. Complementary DNA synthesis was performed using M‐MLV Reverse Transcriptase (Sigma‐Aldrich), oligo dT (Life Technologies), and 2 μg of RNA. Quantitative reverse transcription‐PCR (qRT‐PCR) was performed under universal cycling conditions on LightCycler 480 II (Roche) using TaqMan PCR Mastermix and TaqMan probes (Life Technologies; *LOXHD1* Hs00329848_m1, *FGD4* Hs01030780_m1, *PPIA* Hs04194521_s1). Cycle threshold (CT) values were normalized to that of PPIA. Relative expression levels of the gene of interest were calculated using the ΔΔCT method [39].

### 2.11 Protein extraction and western blot

Protein analysis by western blot was performed as previously described [40]. Primary antibodies used were: CREB1 1:1000 #9197 (Cell Signaling Technology, Danvers, MA, USA); phospho‐CREB1 (Ser133) 1:2000 #9198 (Cell Signaling); FGD4 1:1000 #PA5‐22062 (Invitrogen). Secondary antibodies used were horseradish peroxidase‐conjugated goat anti‐rabbit and goat anti‐mouse (Dako). Peroxidase activity on membranes was detected by enhanced chemiluminescence (Pierce, Thermo Scientific) following manufacturer's instructions. Tubulin 1:5000 #6199 (Sigma‐Aldrich) was used as loading control.

### 2.12 Transient gene silencing

For transient gene silencing, cells were transfected with Lipofectamine 2000 (Invitrogen) following the manufacturer's protocol and using 10 nm of siRNA for *LOXHD1* (SR310375; Origene, Rockville, MD, USA). For *CREB1* transient silencing, cells were transfected with DharmaFECT 1 (Dharmacon) following manufacturer's protocol and using 100 nm of siRNA (SAS1_HS01_0011_6985/CREB1; Merck). After 6 h of transfection, media was changed to remove any remaining Lipofectamine or DharmaFECT 1. As negative control, a non‐targeting siRNA was used (siNT; Sigma‐Aldrich, custom 5′ UAAGGCUAUGAGAGAUAC 3′).

### 2.13 Cell proliferation assay

For viability and proliferation assays, 2500 cells (for Met1 and TC‐252) were seeded in 96‐well plates. At 24 h after seeding, cells were treated with CREB inhibitor 666‐15 (Sigma‐Aldrich) at concentrations of 10, 50, 100, 500, 100, and 1500 nm (DMSO as vehicle control) in culture media. After 24, 48, and 72 h of treatment, the culture media was removed and 100 μL of water‐soluble tetrazolium (WST‐1; Roche) diluted in medium (1:20) was added to each well. After 3 h, cell viability was quantified by spectrophotometry (*λ* = 440 nm) in a PowerWave XS plate reader (Biotek, Winooski, VT, USA).

### 2.14 Boyden chamber assays

For the migration assay, cells were cultured in 6‐well plates (300 000 cells) and treated after 24 h of seeding with either siLOXHD1 (10 nm), siCREB1 (10 nm) or CREB inhibitor 666‐15 (100 and 500 nm). siNT (Sigma‐Aldrich) or DMSO (Sigma‐Aldrich) were used as negative controls, respectively. After 24 h of treatment, cells were harvested as normal and seeded as described previously [41]. Migration assay was stopped after 24 h for Met1A6 or 48 h for TC‐252 and A4573. For the invasion assay, the same approach as for the migration assay was followed, but the Transwells were previously coated with a Matrigel (Corning) layer. For these assays, Transwell Permeable Supports (Corning, NY, USA) membranes with 8.0 μm pores were used. For quantification of migrated/invaded cells, pictures of the membranes were taken and quantified using imagej.

### 2.15 Colony formation assay

Colony assays were performed seeding 500 cells (Met1 and TC‐252) in 6‐well plates and following previously described protocol [41]. After 24 h of seeding, cells were treated once with CREB1 inhibitor 666‐15 (Sigma‐Aldrich) at 10, 50, 100, and 500 nm (DMSO as vehicle control). For quantification of colonies, plates were left to air‐dry and pictures of colonies were taken and quantified using imagej.

### 2.16 Survival analysis

Kaplan–Meier survival analyses were carried out in 166 EwS patients whose molecularly confirmed and retrospectively collected tumors were profiled at the mRNA level by gene expression microarrays in previous studies^98‐101^. To that end, microarray data generated on Affymetrix HG‐U133Plus2.0 or Affymetrix HuEx‐1.0‐st microarrays of the 166 EwS tumors (Gene Expression Omnibus (GEO) accession codes: GSE63157 [42], GSE12102 [43], GSE17618 [44], and GSE34620 [45] provided with clinical annotations were normalized separately as previously described [46]). Only genes that were represented on all microarray platforms were kept for further analysis. Batch effects were removed using the ComBat algorithm [47]. Data processing was done in R. For data analysis on publicly available EWS datasets (GSE17679 and GSE63157), the R2 Genomics Analysis and Visualization Platform (Amsterdam UMC [48]) was used.

### 2.17 Statistical analyses

Statistical analyses for the different omics datasets are described in the respective methods sections. Differences in *in vitro* phenotypic experiments were analyzed using Student's *t*‐test or analysis of variance with Bonferroni's correction. Threshold for significance was *P*‐value <0.05. Statistical analyses were performed using graphpad prism v9.4.0 (GraphPad software, Sant Diego, CA, USA). Statistical differences in graphs as: *P ≤ 0.05, **P ≤ 0.01, ***P ≤ 0.001, ****P ≤ 0.0001.

## 3 Results

### 3.1 Primary tumors and metastases were obtained using a spontaneous metastasis EWS mouse model

**Fig. 1:**
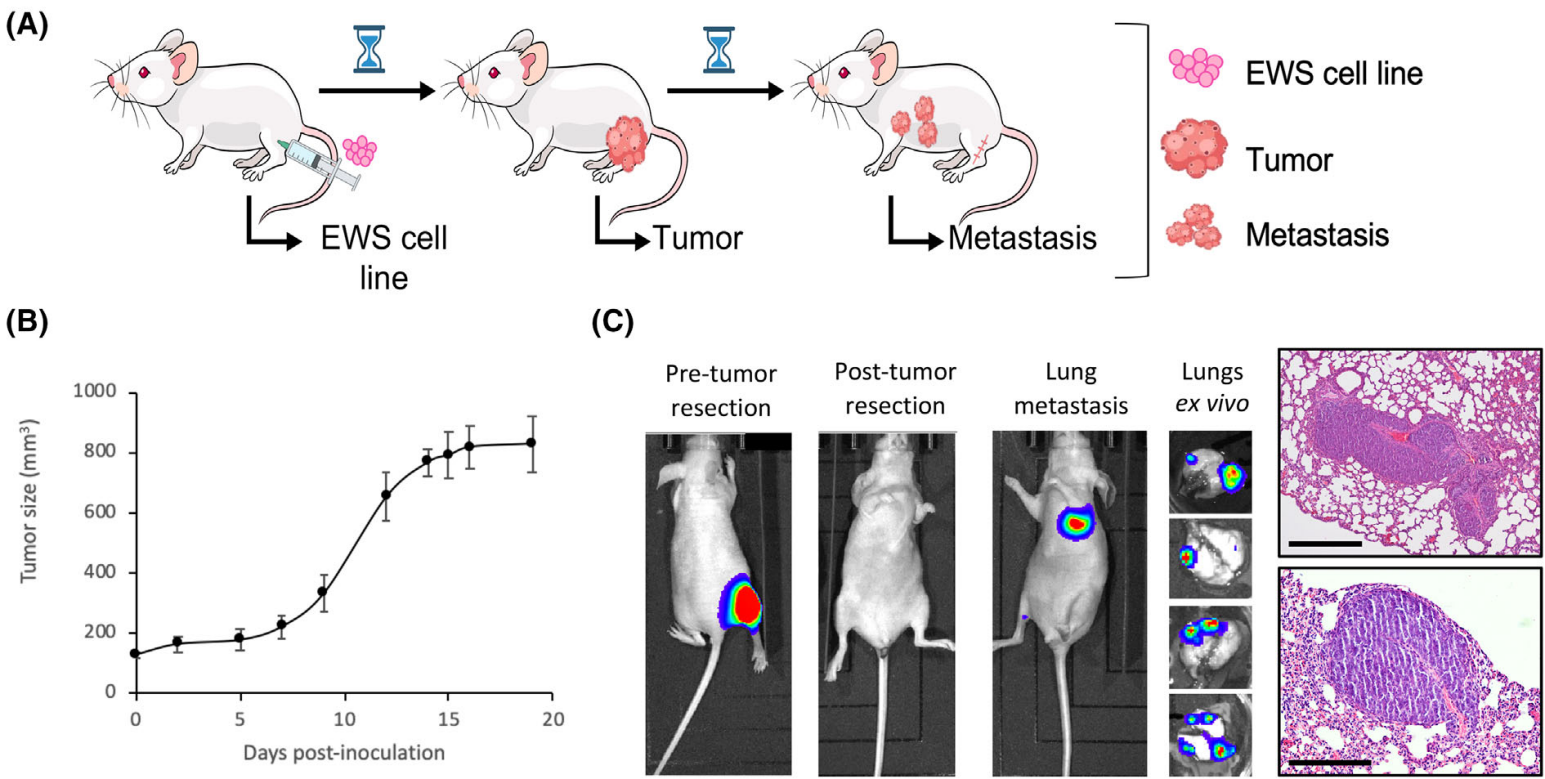
Primary tumors and metastases were obtained from a spontaneous metastasis EWS mouse model. (A) Scheme of the main steps of the whole xenograft orthotopic metastatic assay for A673 and TC‐252 EWS cells. (B) Tumor growth curve of A673Luc cells injected to athymic mice. Error bars indicate SD; *n* = 10 mice. (C) Primary tumor and metastasis detection by luciferase lecture of whole mice, *ex vivo* lungs and immunohistochemistry cuts. A673 model. *n* = 10 mice. Scale bar = 500 μm. EWS, Ewing sarcoma.

Samples of primary tumors and lung metastases were generated by a xenograft orthotopic *in vivo* model [23] (Fig. 1A). Briefly, EWS cells (A673 luciferase‐labeled (A673Luc) or (TC‐252)) were injected into the gastrocnemius muscle of the mice and resected once tumor grew to a size of 800 mm^3^ (Fig. 1B). After surgery, mice could survive for a period long enough to enable the development of distant masses. Metastases were detected at several levels: by *in vivo* lecture of luminescence of the whole mouse, by *ex vivo* luminescence detection or by sight of the lungs, or by histological analysis of the lungs (Fig. 1C). Approximately, 68% of mice developed lung metastasis, that could be detected starting on day 50. No metastases were detected in other tissues than the lungs. For the multi‐omics analyses, a collection of five primary tumors and 12 metastases from the A673 EWS model were used (Table 1). A detailed description of how samples were used for each omics profiling is provided in Table S5. In addition, an extra cohort of A673 and TC‐252 primary tumors and metastases were used for validation studies (Table S5).

**Table 1:** EWS A673 samples analyzed in the multi‐omics approach. Cells injected in mice (A673Luc), primary tumors obtained from the spontaneous metastasis mouse model and lung metastases were obtained from different mice. Samples were analyzed using transcriptomics (Clariom™ D), methylomics (Infinium MethylationEPIC) and proteomics (Phospoho Explorer) arrays. A full description of which samples were used in each assay is described in Table S5. EWS, Ewing sarcoma.

	Total samples available	Clariom™ D assay (RNA)	Infinium MethylationEPIC (DNA)	Phospho Explorer (protein)
Cell line	4*	4	0	0
Primary tumors	5	5	3	3
Metastases	12	12	5	3
Total	21	21	8	6

### 3.2 EWS tumors and metastases have a distinct transcriptomic profile

EWS has a quiet genome, with not many known mutations despite the well characterized translocation involving *EWSR1* gene with different members of the ETS transcription factor family (i.e., *FLI1* or *ERG* amongst others). Therefore, our main focus was to start the omics characterizations with a transcriptomic approach, as it has been suggested that most changes might rely on post‐transcriptional modifications, altering the abundance of RNAs. Using material obtained from the A673 spontaneous metastasis mouse model, we analyzed the transcriptomic profile of five primary tumors and 12 metastases. To study the background profile of these tissues, we included 4 different passages of the cells injected in mice (cell of origin, 2D monolayer A673 cell culture) (Table 1).

**Fig. 2:**
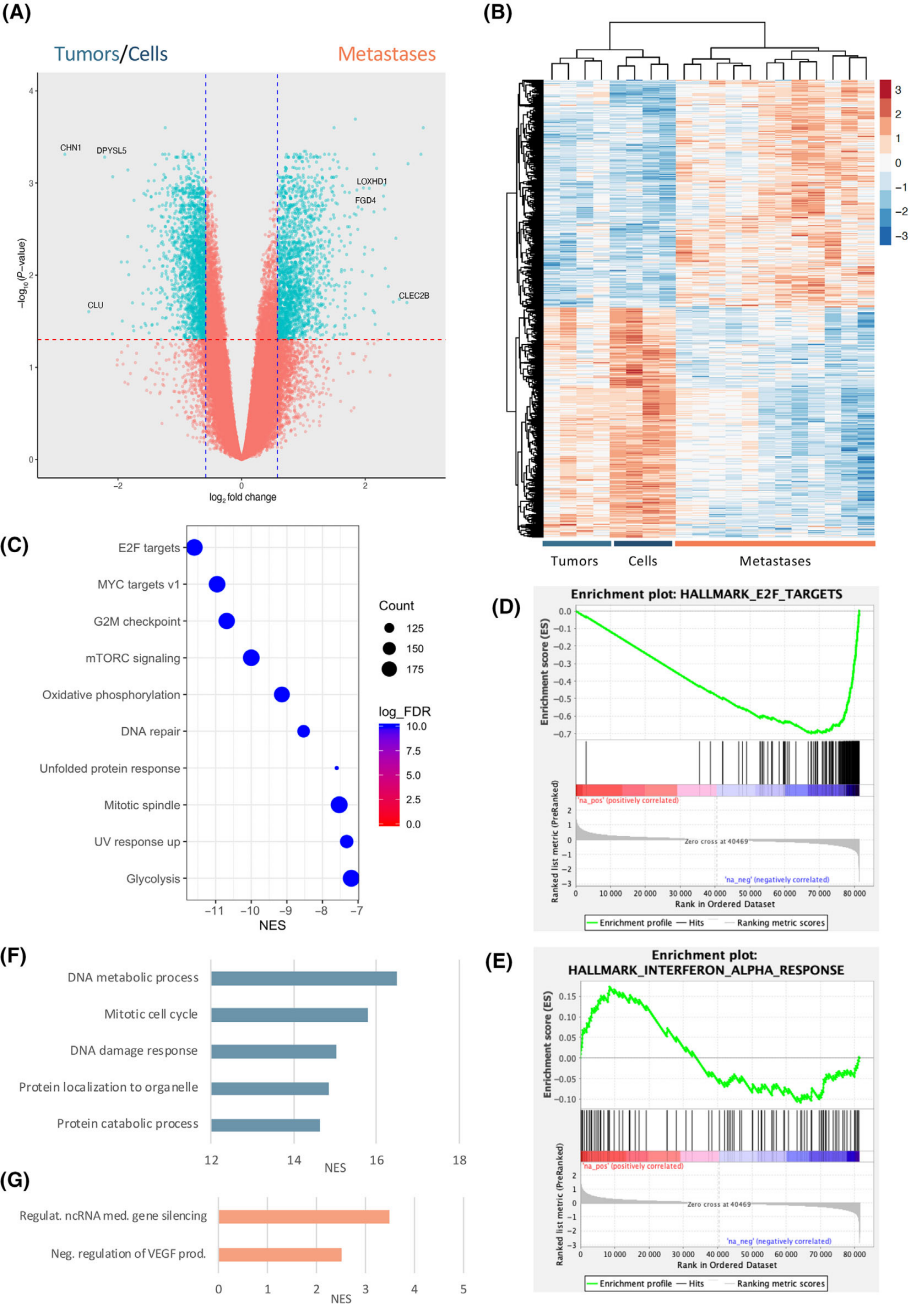
EWS primary tumors and metastases show a different transcriptomic profile. (A) Volcano plot results of differential expression analyses comparing A673 primary tumors and cells (*n* = 4 + 4) vs. metastases (*n* = 12). In blue, all transcripts that are significant (adjusted *P*‐value < 0.05 and absolute (log_2_FC) > 0.58). (B) Heatmap of the differential expression transcripts identified in the comparison between tumor/cells group vs. metastasis. Red and blue range indicating higher and lower expression values, respectively. (C–G) GSEA pathway analysis of transcriptomic EWS data. Hallmark database identified different gene sets enriched in cells/tumors (top10, C), including E2F targets (D) and only one gene set enriched in metastasis (IFNα response, E). Gene ontology (biological process) database showed enrichment for different datasets in cells/tumors (top5, F) and only two in metastasis (G). Statistics for selection: −log_10_(FDR *P*‐value) < 0.05. EWS, Ewing sarcoma; FDR, false discovery rate; GSEA, gene set enrichment analysis.

After quality control, one of the primary tumors was excluded, leaving 20 samples for the downstream analyses (Table S5). Although all tissues used are originated from the same EWS cell line, cellular modulations within the *in vivo* model led to changes in their transcriptomic profile, being able to distinguish between cells, tumors and metastases (Fig. S1). Based on this, our approach was to explore the differences between EWS primary tumors and metastases to understand the biological changes cancer cells overcome to become metastatic. As EWS cell lines clustered near tumors, we grouped these two conditions to increase the power of the analysis. Therefore, a differential expression analysis was performed comparing EWS metastatic samples (*n* = 12) vs. primary tumors and cells (*n* = 4 + 4). A total of 4139 differentially expressed genes (DEG) were identified (fold change (FC) > 1.5, adjusted *P*‐value <0.05), with 2075 enriched in metastases and 2064 in the tumors/cells group (Fig. 2A,B). It is important to mention that these DEG include unannotated transcripts and probes, which were discarded for downstream pathway analyses as they would not provide any insight. Out of the 2075 DEG in metastases, 1583 were unannotated transcripts (76.3%), leaving only 492 DEG. In contrast, only 12.1% of DEG from the tumors/cells group were unannotated transcripts (249/2064), leaving a list of 1815 DEG. This difference in the expression profile adds another layer of complexity when investigating the profile of EWS metastases.

Several pathway analyses were performed to understand the biological processes each sample group was exploiting. Using pre‐ranked GSEA, we explored the differences between metastatic and tumor/cell samples. Of interest, when investigating hallmark gene sets, the profile of tumors/cells group was associated to important cell cycle mechanisms (i.e., E2F targets, G2/M checkpoint; Fig. 2C), being E2F targets at the top (NES = −11.6, FDR *P*‐value <0.001; Fig. 2D). Metastatic samples were only significantly associated to IFNα response (NES = 2.0, FDR *P*‐value = 0.005; Fig. 2E). When investigating GO datasets (Biological Process), an enrichment for cell cycle processes in tumors/cells (top5; Fig. 2F) and regulation of ncRNA or VEGF in metastases (Fig. 2G) were detected.

With these results, we confirm that a cellular modulation occurs within the development of spontaneous metastases in mice that lead to changes in the transcriptomic expression profile, even though all samples come from the same cell of origin (A673). The changes observed in the pathway analyses confirm the activation of distinct mechanisms between metastatic and tumor samples. To verify the results obtained, we performed pathway analyses using the IPA (Qiagen) software. Here, we identified similar pathways to be enriched in this dataset (i.e., *E2F1* or cell cycle progression) (Fig. S2A). One of the analyses that IPA provides is to investigate causal relationships associated with our dataset with a focus on upstream analyses and putative regulators. When doing this, 425 different master regulators of causal networks were identified. Some of these were only regulating themselves (i.e., *CKAP2L* or *NUPR1*) but other were interconnected with many different genes (i.e., *FGF1* to 70 regulators or *p38 MAPK* to 85 regulators). Some of these master regulators were predicted to be activated whereas others were inhibited. Of interest, *PP1R1B* was the master regulator with highest *P*‐value (3.16 × 10^−20^). As can be seen in Fig. S2B, this regulator is linked to different signaling cascades, some of which have changes in expression on our dataset (reported in red/green) and some have predicted activation/inhibition although not detected in our transcriptomic data (in orange/blue respectively). The results obtained from the different pathway analyses on our transcriptomic data alongside the clustering of the DEG indicate that EWS metastases have a distinct expression profile than their primary tumors and cell lines.

When focusing on specific transcripts that were significantly overexpressed in metastases compared to tumors and cells, we found Lipoxygenase Homology PLAT Domains 1 (*LOXHD1*; log_2_FC 2.1; adj *P*‐value 0.001) and *FGD4* (log_2_FC 1.9; adj *P*‐value 0.002) amongst others. Following our hypothesis, these transcripts might be important for the process of metastasis in EWS, as they are overexpressed in EWS metastases compared to the primary tumors and cell lines that these come from.

### 3.3 Methylomics analysis identifies a distinct profile between EWS primary tumors and metastases

**Fig. 3:**
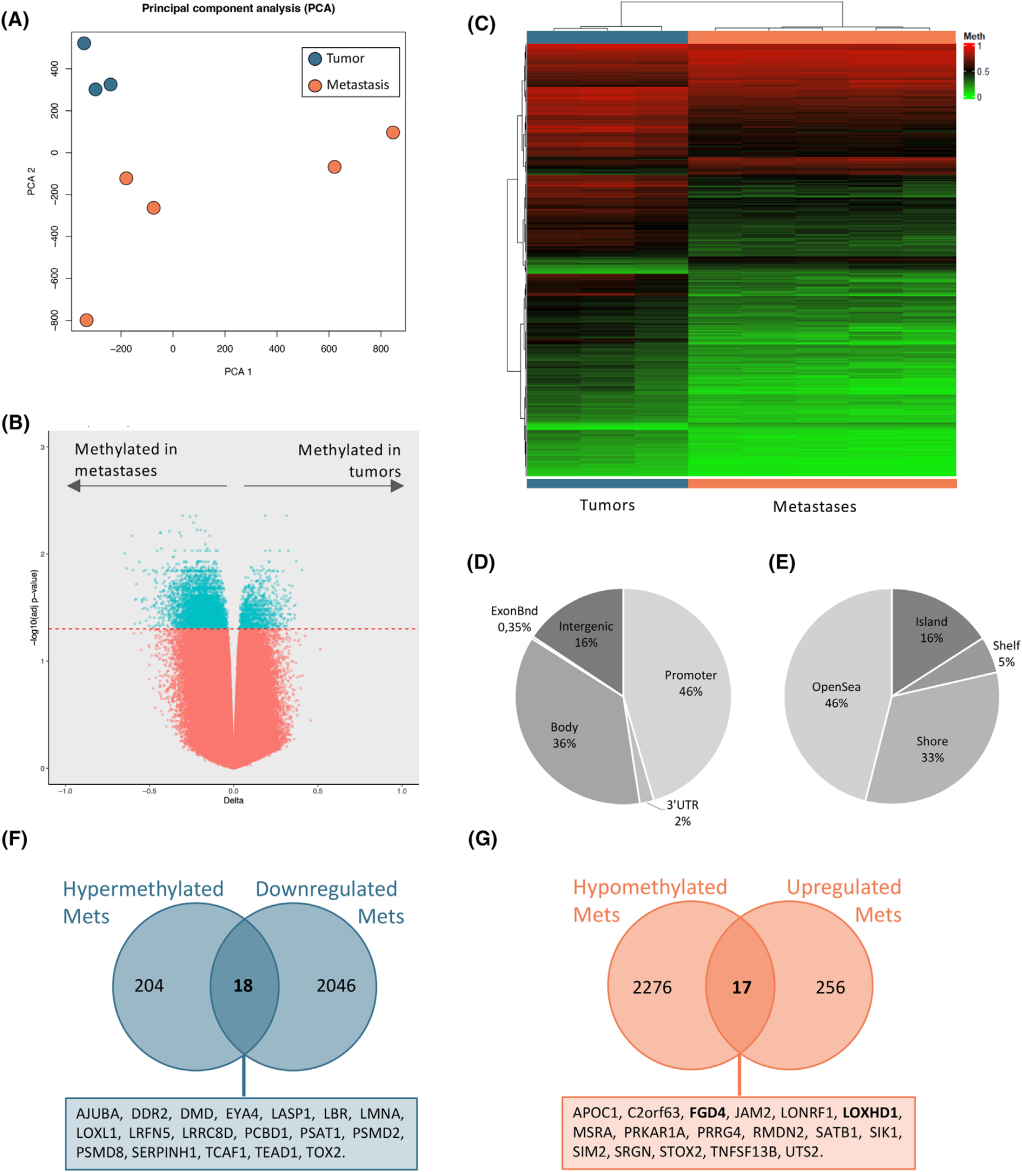
Tumors and metastases show a distinct methylation profile. (A) PCA of A673 EWS primary tumors and metastases show a different methylation profile. (B) After quality control, a total of 3375 genes with 5964 differentially methylated CpGS (FDR adjusted *P*‐value < 0.05) were identified between tumors and metastases. In turquoise, genes with significant differences. (C) Hierarchical clustering heatmap of the differentially methylated CpGs (DMCpGs; FDR adjusted *P*‐value < 0.05) obtained between metastases and primary tumors. Genomic distribution of 5964 DMCpGs between primary tumors and metastases, in relation to (D) gene region and (E) CpG context. (F, G) Genes present in the overlap between (F) promoter hypermethylated and transcripts downregulated in metastases, or between (G) promoter hypomethylated and transcripts upregulated in metastases. In bold, two genes of interest validated later on. Samples analyzed *n* = 8. EWS, Ewing sarcoma; FDR, false discovery rate; PCA, principal component analysis.

To better understand the process of metastasis in EWS, we cannot focus on transcriptomic data only. Hence, DNA from three paired primary tumors and metastases (*n* = 3 + 5) derived from our A673 spontaneous metastasis mouse model (Table 1) was extracted and profiled using the Infinium MethylationEPIC (EPIC) arrays (Illumina). This methylation screening array includes more than 850 000 CpG sites in the human genome, providing a unique opportunity to decipher the methylation changes in EWS metastases compared to tumors. After quality control, PCA showed that PCA2 was separating metastatic samples from tumors (Fig. 3A). Amongst all the CpGs analyzed, we obtained 5964 significant (*P*‐value <0.05; FDR adjusted *P*‐value <0.05) differentially methylated CpGs (DMCpGs) between metastases and primary tumors, which linked back to 3375 different genes (Fig. 3B). A hierarchical clustering analysis with the 5964 DMCpGs displayed a DNA methylation profile able to clearly differentiate metastases from primary tumors (Fig. 3C). These DMCpGs were distributed in several regions of the genome (Fig. 3D) with different density of CpGs (Fig. 3E), including promoters and CpG islands (CpGIs). Interestingly, in comparison with primary tumors, the promoter CpGIs of metastatic samples were hypomethylated in 2276 genes and hypermethylated in 204 genes.

In cancer, aberrant methylation of promoter CpGIs is usually linked to changes in gene expression, with a negative correlation between methylation and gene expression [49]. Therefore, we evaluated the association between the methylation changes detected in the CpGIs of promoters with the gene expression obtained in the transcriptomic analyses of EWS (Fig. 3F,G). This comparison would allow to shed some light into the mechanisms by which the expression of some of these genes is regulated. In line with this, metastatic samples presented 18 hypermethylated and downregulated genes (Fig. 3F), and 17 hypomethylated and upregulated genes (Fig. 3G). To validate the results obtained in the methylomics EWS dataset, two genes that were hypomethylated in metastases and which were present amongst the DEG in metastases (*LOXHD1* and *FGD4*) were chosen, as both have been associated to tumorigenic characteristics in other cancers [50, 51, 52, 53, 54, 55, 56]. Pyrosequencing analysis of the promoter CPGIs of both genes confirmed lower methylation levels at all A673 EWS metastases compared to the tumor samples (Fig. S3A,B). To validate this, pyrosequencing analysis was also performed on metastases and tumors from the spontaneous metastasis TC‐252 mouse model for *LOXHD1* (Fig. S3A). These results confirm that a distinct methylation status between tumors and metastases could be behind the differential expression of these genes. To validate these results on *LOXHD1*, we treated A673 and TC‐252 cells with Decitabine, a nucleoside analogue that inhibits DNA methylation [57]. Interestingly, we observed a reduction on the methylation levels on *LOXHD1*'s promoter for both cell lines (Fig. S4A), accompanied by an increase in *LOXHD1* expression in A673 (1.0 vs. 9.3, adj *P*‐value = 0.0002; Fig. S4B). These results suggest that indeed, *LOXHD1*'s expression in EWS metastatic samples could be regulated by the methylation status of its promoter.

### 3.4 Proteomics in EWS samples identify CREB1's activation to be enriched in EWS metastases

**Fig. 4:**
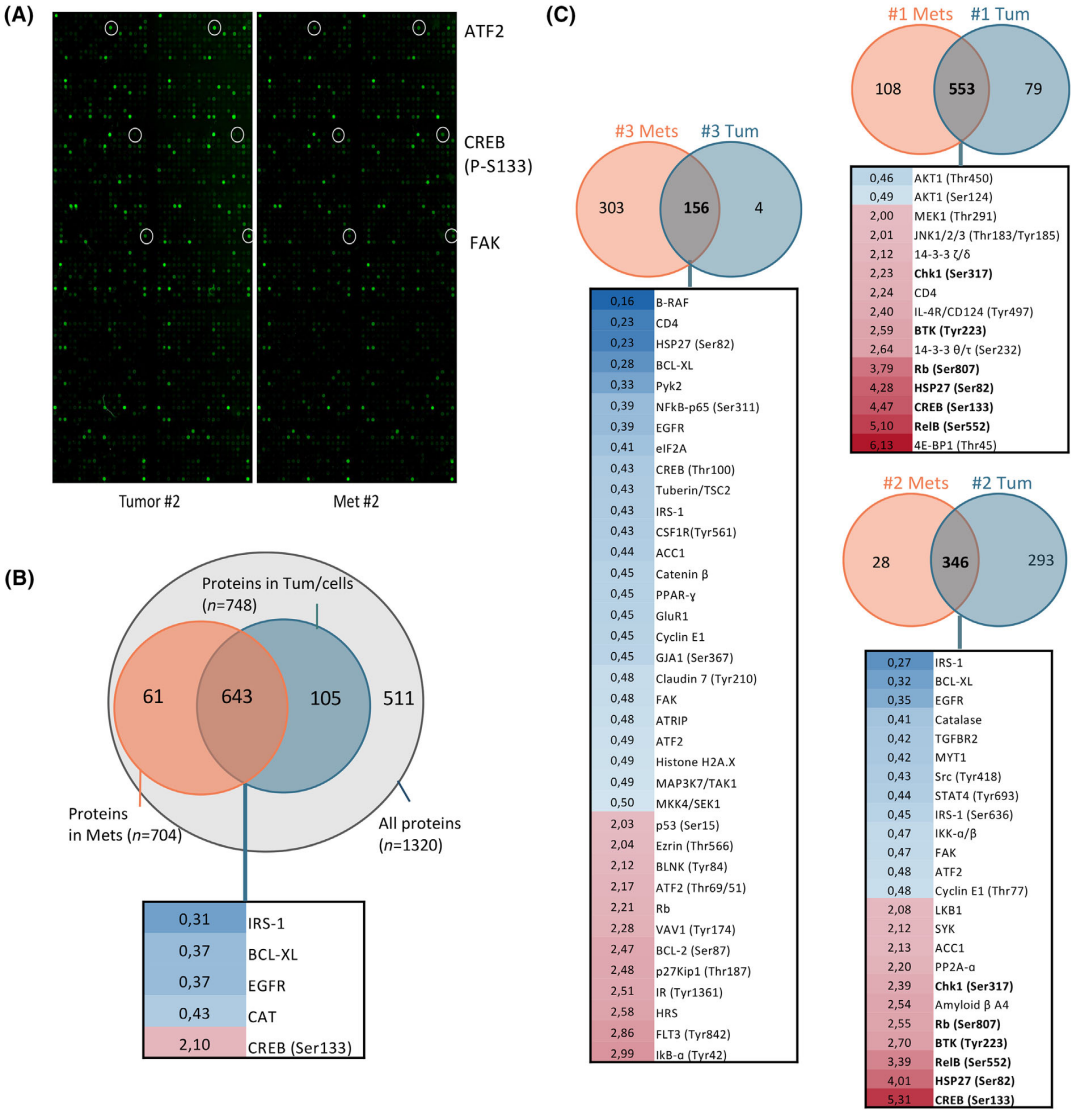
Proteomic profiling of EWS primary tumors and metastases reveals a distinct protein expression profile. (A) Example of a tumor (left) and a metastasis (right) chip from the Phospho Explorer array. Green fluorescent intensity indicative of protein expression (lighter = more expression). (B) Venn diagram indicating, from the total number of proteins present in the array (1320), which proteins were detected in each sample group. In the overlap (proteins detected in both sample groups), the list of proteins that passed the selection criteria (0.5 < FC >2; blue = tumors, red = metastases). (C) Comparison of each pair of A673 tumor‐metastasis, indicating the proteins detected in each subset and those shared. From these, the list and FC expression values are shown for those that passed selection criteria. In bold, those shared in 2/3 metastases. Red = higher expression in metastases; Blue = higher expression in tumors. Samples analyzed; *n* = 6. EWS, Ewing sarcoma; FC, fold change.

After gaining some insight into the transcriptomic and methylomic profiles of EWS tumors and metastases, we wanted to include a third layer of data: proteomics. Expression of genes is fundamental, but on many occasions, small transcriptomic changes or post‐translational modifications lead to proteins being differentially expressed in different samples and contexts. For this, three paired tumors and metastases from the A673 EWS spontaneous metastasis mouse model (Table 1) were profiled using a Phospho Explorer Antibody Array. This array includes more than 1300 antibodies associated to multiple proteins important to cell signaling pathways (Fig. 4A).

Through this, a total of 809 proteins (native and phosphorylated forms) were detected in EWS samples (Fig. 4B). The remaining 511 proteins of the array were not detected in our samples, which might suggest that their expression is too low to be detected or that they are not fundamental for EWS biology. In addition, a proportion of the proteins included in this analysis were only detected in tumors (*n* = 105) or in metastases (*n* = 61), indicating the differential profile of both sample groups (Fig. 4B). From the 643 proteins detected in both sample groups, the phosphorylated states of some protein were elevated in metastases (FC > 2; phospho‐CREB1 (Ser133)) and the expression of other proteins in tumors (FC < 0.5: IRS‐1, BCL‐XL, EGFR, CAT) (Fig. 4B). Due to the nature of this assay, and the different expression values across the 3 paired samples, it was not possible to obtain significant differences, except for IKKα/β (*P*‐value = 0.04, FC = 1.7). Moreover, seven other proteins had significant differences (*P*‐value <0.05) in their expression between tumors and metastases, but their FC values were outside our criteria (in FC order: E2F1, ErbB4, FOXO3A, MSK1, ATF2, Chk1, and LKB1). We then performed paired comparisons, where each tumor sample was compared to its metastasis, in order to identify proteins that were enriched in each pair. As shown in Fig. 4C, each comparison resulted in a different profile between tumors and metastases. Proteins listed under each Venn diagram indicate those that have FCs over the specified threshold (<0.5 and >2). In contrast to doing the analyses merging all metastases vs. all tumors, there were no proteins with distinct expression profile shared across the three comparisons. Only the phosphorylation state of six proteins (RelB (Ser552), Rb (Ser807), BTK (Tyr223), Chk1 (Ser317), CREB (Ser133), and HSP27 (Ser82); in bold Fig. 4C) had higher levels in 2/3 metastatic samples. Of these, only phosphorylated CREB1 (Ser133) was detected as well when comparing all samples together (Fig. 4B), suggesting that phosphorylation of CREB1 at Ser133 is associated to EWS metastases. When comparing the list of proteins identified in metastases (*n* = 704) and those DEG from metastases (*n* = 273), only two were present in the overlap: ERBB4 and CASP1. However, it is important to mention that the phosphoarray used here is focused on signaling proteins (native and phosphorylated form), and therefore, all those proteins not relevant here would not be present in the overlap (i.e., LOXHD1 or FGD4). These proteomics results suggest that, although paired tumors and metastases have a distinct proteomic profile, there are differences in the pathways detected across the EWS samples investigated.

### 3.5 CREB1 putative target genes amongst genes overexpressed and hypomethylated in EWS metastases

**Fig. 5:**
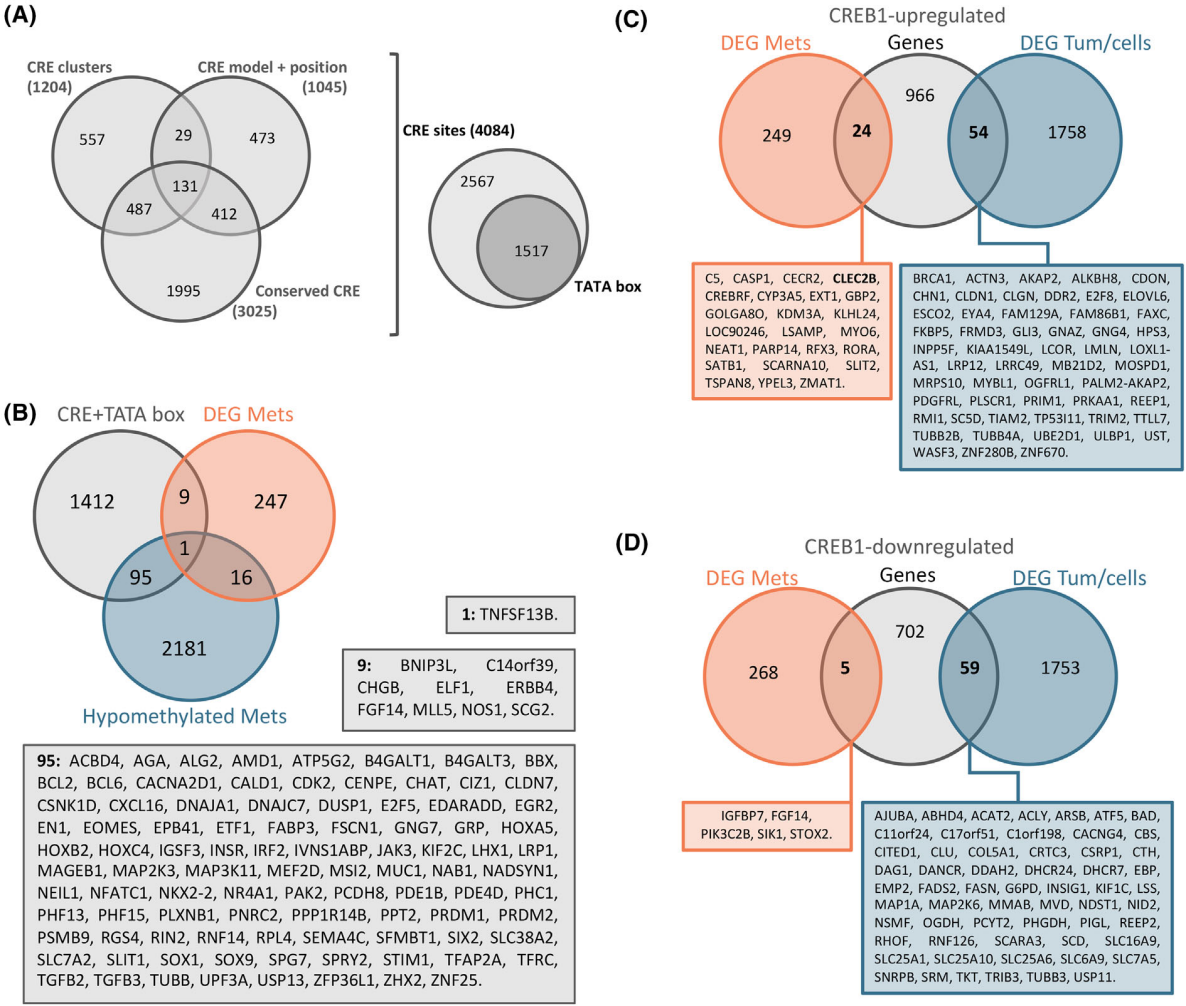
Integration of EWS transcriptomic and methylomic data with putative CREB1‐binding sites. (A) Identification of putative CREB‐binding sites based on *in silico* analysis by Zhang et al. [58]. Only sites with CRE regions (based on *in silico* analysis) and TATA boxes in the near region (*n* = 1517) are used. *n* = 4084 CRE sites analyzed. (B) Overlap between CRE sites with TATA boxes (*n* = 1517), genes overexpressed in metastases (*n* = 273), and genes hypomethylated in metastases (*n* = 2293) is shown, with lists of genes identified in each overlap. (C) A list of target genes upregulated by CREB1 expression (from KO – rescued CREB1 models [59]; *n* = 1044) was compared to our EWS transcriptomic data (*n* = 273 and *n* = 1812). (D) A list of target genes downregulated by CREB1 expression (from KO – rescued CREB1 models [59]; *n* = 766) was compared to our EWS transcriptomic data (*n* = 273 and *n* = 1812). The genes identified in the overlaps are listed underneath. CRE, cAMP response element; DEG, differentially expressed genes; EWS, Ewing sarcoma; KO, knock out.

The phosphorylation of CREB1 at Ser133 was identified to be enriched in metastatic EWS samples compared to tumors. As this is a well‐known transcription factor involved in multiple signaling pathways, we decided to study the putative role of CREB1 in regulating the profile of EWS metastatic samples. For this, we used the *in silico* results from Zhang et al. [58], where they describe all the genes with cAMP response elements (CRE) conserved sites (*n* = 3025), those with positional conserved CRE sites across different species (*n* = 1045) and those genes with clusters of CRE (*n* = 1205), obtaining a list of 4084 genes that fulfilled the different criteria (Fig. 5A). Then, they identified which of those 4084 genes had a TATA box on the promoter, reducing the list to 1517 (Fig. 5A). As the focus of this study is to find targets enriched in EWS metastases, we combined this list of putative *CREB1*‐target genes (*n* = 1517) with transcripts enriched in EWS metastases (transcriptomics data, *n* = 273) and genes hypomethylated in EWS metastases (methylomic data, *n* = 2293) (Fig. 5B). As shown, there is some overlap between the different lists. Of interest, there is one gene (*TNFSF13B*, also known as *CD257* or *BAFF*) which appears in the three lists, suggesting that its overexpression in EWS metastases could be regulated by *CREB1*. Moreover, there are nine genes which expression is enriched in metastases (transcriptomic data) and 95 genes which are hypomethylated in metastases (methylomic data) that fulfill all CRE‐associated criteria (Fig. 5B), suggesting a putative regulation of their expression via CREB1 signaling. Of interest, amongst the CRE‐TATA gene list (genes regulated by *CREB1*), we identified *C/EBPβ*. When investigating the CpG with different methylation status on the *FGD4* promoter (cg22431093), we identified binding sites for this transcription factor (UCSC Genome Browser GRCh37). This suggests that *C/EBPβ* could be regulating the expression levels of *FGD4* depending on the methylation of its promoter region, and that *C/EBPβ* itself is regulated by *CREB1* by a CRE‐binding site in its promoter.

Nonetheless, a recent publication by Zheng et al. [59] explores the possibility of many *CREB1* target genes regulated independently of cAMP and CRE sites. To test this, they performed *CREB1* knock‐out (KO) and *CREB1*‐rescue (on KO cells) on HeLa cells and compared their expression using RNA‐seq. We used their DEG lists of putative *CREB1*‐target genes to compare them to our transcriptomic EWS results. Zheng et al. [59] identified 1044 putative *CREB1*‐target genes upregulated in cells expressing *CREB1* (wild type vs. KO or rescue vs. KO). Of these, 24 were present amongst our EWS metastasis‐upregulated transcripts (*n* = 273) (Fig. 5C) and 54 amongst EWS tumor/cell‐upregulated transcripts (*n* = 1812) (Fig. 5C). When evaluating the putative *CREB1*‐target genes downregulated in cells expressing *CREB1* (down in rescue and wild type vs. KO), five were shared with our EWS metastases‐associated DEG (Fig. 5D) and 59 with our EWS tumor/cell‐associated DEG (Fig. 5D). Overall, these results indicate that *CREB1* target genes in EWS could be both regulated by cAMP and CRE‐dependent manner (Fig. 5B) and a cAMP and CRE‐independent manner (Fig. 5C,D). Further research needs to be put into the different CREB1‐mediated signaling cascades that could be modulating the behavior of cancer cells, as both could be regulating important mechanisms in EWS metastasis.

### 3.6 The transcription factor CREB1 regulates the metastatic phenotype of EWS

**Fig. 6:**
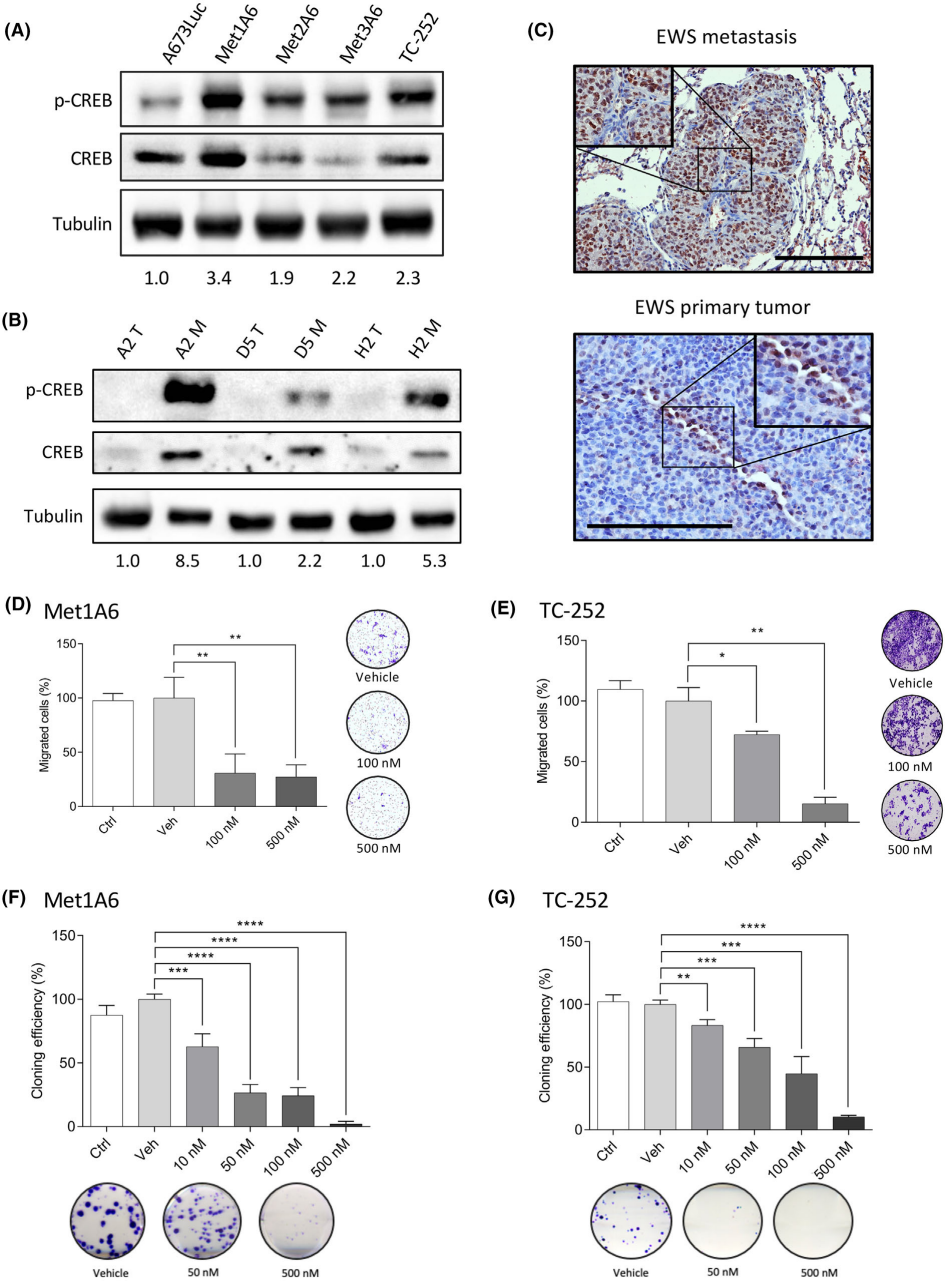
CREB1 has a role in the metastatic profile of EWS samples. (A, B) Protein levels of CREB1 and phospho‐CREB1 (Ser133) evaluated by western blot in (A) three metastasis‐derived primary cell lines from *in vivo* lung A673Luc metastases and (B) paired primary tumors and metastases from the TC‐252 *in vivo* model (T = tumor; M = metastasis). Tubulin as loading control. Numbers indicative of phospho‐CREB1 ratio normalized to tubulin, respective to A673Luc or paired primary tumor respectively. (C) Protein levels of phospho‐CREB1 (Ser133) evaluated by immunohistochemistry in EWS metastases and primary tumors from the A673 spontaneous metastasis mouse model. Expression as brown staining. Scale bar = 500 μm (top) and 200 μm (bottom). (D, E) Migration assay using Boyden chamber principle on (D) Met1A6 and (E) TC‐252 cells treated with CREB1 inhibitor 666‐15. Values from two independent repeats, at 24 and 48 h of migration respectively. Images of representative membranes. (F, G) Colony formation assay on (F) Met1A6 and (G) TC‐252 cells treated with CREB1 inhibitor 666‐15. Values from two independent repeats. Images of representative wells. ANOVA test. Error bars indicate standard deviation of the mean (SEM). Statistical differences as: *P ≤ 0.05, **P ≤ 0.01, ***P ≤ 0.001, ****P ≤ 0.0001. Three independent replicates were performed for each experiment. EWS, Ewing sarcoma.

On the proteomic analysis, CREB1 (with its phosphorylated form on Ser133) stood up as putative driver of EWS metastases (Fig. 4). *In silico* analysis identified an overlap between putative CREB1 targets and our transcriptomic and methylomic A673 EWS datasets (Fig. 5). Therefore, we decided to further validate its role in EWS metastatic cells using *in vitro* experiments. First, we evaluated the expression of CREB1 and phospho‐CREB1 (Ser133) in A673Luc metastasis‐derived primary cultures originated from *in vivo* A673 metastases (Met1‐3A6; Fig. 6A). Indeed, expression of phospho‐CREB1 was increased in metastatic cell lines compared to established cell cultures (A673Luc). This was also seen in paired primary tumors and metastases from the TC‐252 *in vivo* samples (Fig. 6B), where expression of phospho‐CREB1 was increased in metastases. Using histology sections from A673 EWS primary tumors and metastases used in our omics profiling, we identified an upregulation of phospho‐CREB1 in EWS metastases compared to primary tumors, where only a few cells showed positive staining (Fig. 6C). Both western blot and immunohistochemistry results confirm that phospho‐CREB1 is indeed upregulated in EWS metastases in both EWS models. To study if CREB1 is regulating important metastatic functions in EWS cells, we decided to inhibit CREB1 signaling cascade using 666‐15, a potent inhibitor of CREB's activity by blocking the KID‐KIX interaction [60]. When treating Met1A6 and TC‐252 cells with different concentrations of 666‐15 inhibitor, we saw a low decrease in CREB1 and phospho‐CREB1's expression, which was more visible for TC‐252 (Fig. S5A). Although 666‐15 inhibits the interaction between CREB1 and CBP/p300, it might be indirectly affecting its expression levels via other signaling mechanisms, thus affecting CREB1's canonical expression too. A decrease in phospho‐CREB1 expression was also visible after transient silencing of CREB1 (Fig. S5B).

If CREB1 is indeed regulating genes associated to the metastatic phenotype of EWS cells, it is fundamental to study how inhibition of CREB1's signaling cascade affects migration ability of EWS metastatic cells. After treatment with 666‐15 on Met1A6 (Fig. 6D) and TC‐252 (Fig. 6E) cells for 24 h, we evaluated the migration ability using the Boyden chamber assay. As shown, 666‐15 inhibits Met1A6 ability to migrate at 100 nm (69% reduction vs. vehicle, *P*‐value = 0.004). For TC‐252, this inhibition is less potent, as 100 nm only reaches a 38% reduction (*P*‐value = 0.03) but is stronger at 500 nm (85% reduction vs. vehicle, *P*‐value = 0.004). To confirm that these results were not caused by an effect of 666‐15 on the proliferation ability of EWS cells, we studied the proliferation of Met1A6 and TC‐252 when treated with different doses of 666‐15 (Fig. S5D). Met1A6 proliferation is not affected until reaching high concentrations (1500 nm at 48–72 h), which could be toxic for EWS cells. However, TC‐252 proliferation is affected at lower doses of inhibitor, especially at 72 h. Although migration experiments are performed only after 24 h of treatment, and therefore, proliferation effects should be independent on migration differences, it is important to take it into consideration if doing longer time‐points. To further validate the role of CREB1 in EWS cells, we evaluated the migration ability of TC‐252 cells after genetic silencing, obtaining a reduction of 39% compared to siNT control (100 ± 10% vs. 61 ± 10%, *P*‐value <0.0001; Fig. S5C).

Another important characteristic of cancer cells is their colony forming ability. Therefore, we studied how this phenotype was affected in Met1A6 (Fig. 6F) and TC‐252 (Fig. 6G) after treatment with a range of 666‐15 inhibitor (10–500 nm). After a single treatment, the colony forming ability was highly impaired, seeing results at low concentrations for Met1A6 (50 nm 34% reduction, *P*‐value <0.0001; 500 nm 90% reduction, *P*‐value <0.0001) and TC‐252 (50 nm 73% reduction, *P*‐value <0.0001; 500 nm 98% reduction, *P*‐value <0.0001) compared to their respective vehicle controls. Altogether, these results suggest that CREB1 could act by mediating the activation of genes important for EWS metastatic profile, and thus deserves further attention in future studies.

### 3.7 LOXHD1 is overexpressed in EWS metastases and plays a role in migration

**Fig. 7:**
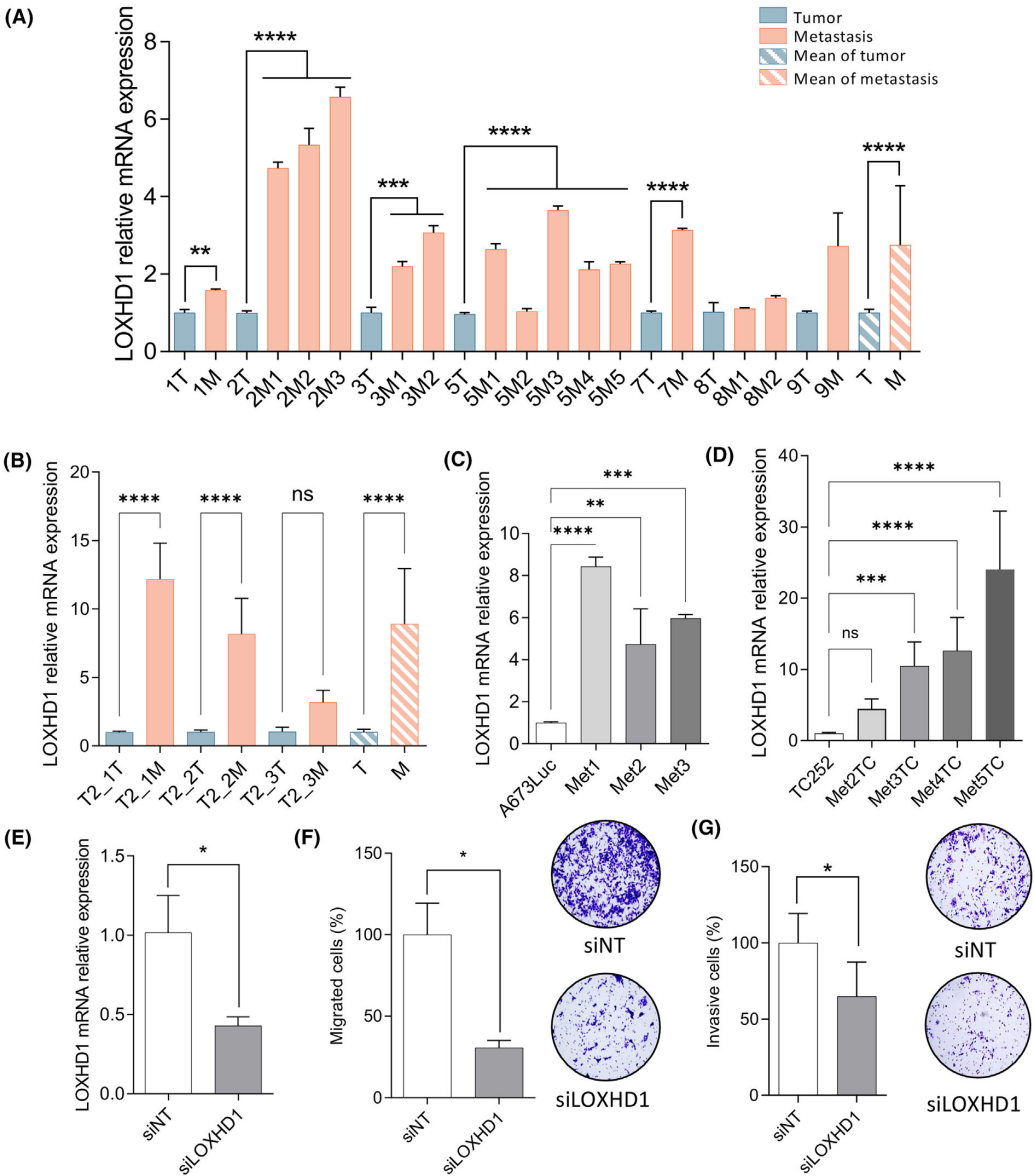
EWS metastatic profile is partly regulated by LOXHD1 expression. (A) qPCR validation of LOXHD1 expression on EWS A673 tumors (blue) and metastases (orange) on samples used for the array (1T‐3T, 5T) and extra cohort of EWS A673 samples (7T‐9T). Mean expression values for tumors (T) and metastases (M) plotted (stripped). (B) qPCR validation of *LOXHD1* expression on EWS TC‐252 tumors (blue) and metastases (orange) on three different paired samples. Mean expression values for tumors (T) and metastases (M) plotted (stripped). (C, D) Expression of *LOXHD1* by qPCR on (C) primary metastasis‐derived primary cell lines from *in vivo* lung A673Luc model and (D) TC‐252 *in vivo* model. qPCR values normalized to housekeeping gene (*PPIA*) and expressed in comparison to paired tumor/cell line expression. (E) Transient silencing of *LOXHD1* in A4573 EWS cell line after 48 h (10 nm). (F, G) Migration ability (F) and invasion ability (G) of A4573 EWS cells is impaired when *LOXHD1* is silenced (transient silencing) after 72 h of silencing. Results based on migrated/invasive cells after 48 h on Boyden chamber assay. Pictures of the bottom of chambers and their quantification (imagej). Three independent replicates were performed for each experiment. ANOVA test. Error bars indicate SEM. Statistical differences as: *P ≤ 0.05, **P ≤ 0.01, ***P ≤ 0.001, ****P ≤ 0.0001. EWS, Ewing sarcoma.

Based on the transcriptomic and methylomic data, we also decided to study the expression of *LOXHD1* in EWS samples. *LOXHD1* is a gene that encodes for a protein associated to hearing loss [54, 56] but has recently been linked to EWS cell motility and metastasis [52]. First, the expression of *LOXHD1* was studied by qPCR on the same A673 *in vivo* samples used for the transcriptomic array (Fig. 7A), seeing an overexpression in metastatic samples compared to paired tumors in all cases (4/4; colored bars up to 5T‐5M). To further validate this profile, an extra cohort of EWS tumors and metastases obtained from the A673 spontaneous metastasis mouse model was analyzed (colored bars up to 9T‐9M), seeing again an overexpression of *LOXHD1* in metastases. When considering the overall EWS profile, the expression of *LOXHD1* in metastatic samples was significantly increased compared to paired tumors (3 ± 0 vs. 1 ± 0, *P*‐value <0.0001; stripped bars) (Fig. 7A). This enrichment on metastatic samples was also detected in a cohort of *in vivo* TC‐252 paired tumors and metastases, corroborating that these results are not A673‐dependent (Fig. 7B). Using primary cultures obtained from A673Luc and TC‐252 lung metastases of both *in vivo* models (low passages), we further studied the expression of LOXHD1 in metastasis‐derived primary cultures compared to the cell of origin (A673Luc or TC‐252 respectively; cells injected in mice) (Fig. 7C, D). Again, *LOXHD1* was overexpressed in the different A673 metastasis‐derived primary cultures compared to the control cells (A673Luc 1 ± 0 vs. Met1A6 8 ± 0 (*P*‐value <0.0001), vs. Met2A6 5 ± 1 (*P*‐value = 0.02), vs. Met3A6 6 ± 0 (*P*‐value <0.0001)). This was also observed in TC252‐derived metastatic cultures (TC‐252 1.0 ± 0.1 vs. Met3TC 10.5 ± 3.4 (*P*‐value = 0.0002), vs. Met4TC 12.6 ± 4.7 (*P*‐value <0.0001), vs. Met5TC 24.0 ± 8.2 (*P*‐value <0.0001); Fig. 7D). Altogether, these data confirm that the profile identified through transcriptomics in a subset of EWS *in vivo* samples correlates with that seen in other *in vivo* samples and *in vitro* cultures from different EWS cell lines.

We then transiently silenced the expression of *LOXHD1* in EWS‐metastatic established cell line A4573, reaching a 57% decrease in *LOXHD1* mRNA expression (siNT 101% ± 13, siLOXHD1 43% ± 3, *P*‐value = 0.013; Fig. 7E). To study if LOXHD1 could be important for the EWS metastatic phenotype, we silenced its expression using siRNAs and studied its ability to migrate and invade using the Boyden chamber assay. A4573 EWS metastatic cells with *LOXHD1* knock‐down showed a decreased migration ability compared to mock siRNA (siNT 100% ± 19, siLOXHD1 30% ± 4, *P*‐value = 0.025; Fig. 7F). This was also observed when evaluating their invasive phenotype upon *LOXHD1* silencing (siNT 100 ± 19% %, siLOXHD1 64 ± 22% %, *P*‐value = 0.048; Fig. 7G). These results confirm that LOXHD1 is indeed an essential factor in EWS metastatic phenotype, as two independent groups using two different approaches have come to the same conclusions [52].

### 3.8 EWS metastatic lesions present high FGD4 expression

**Fig. 8:**
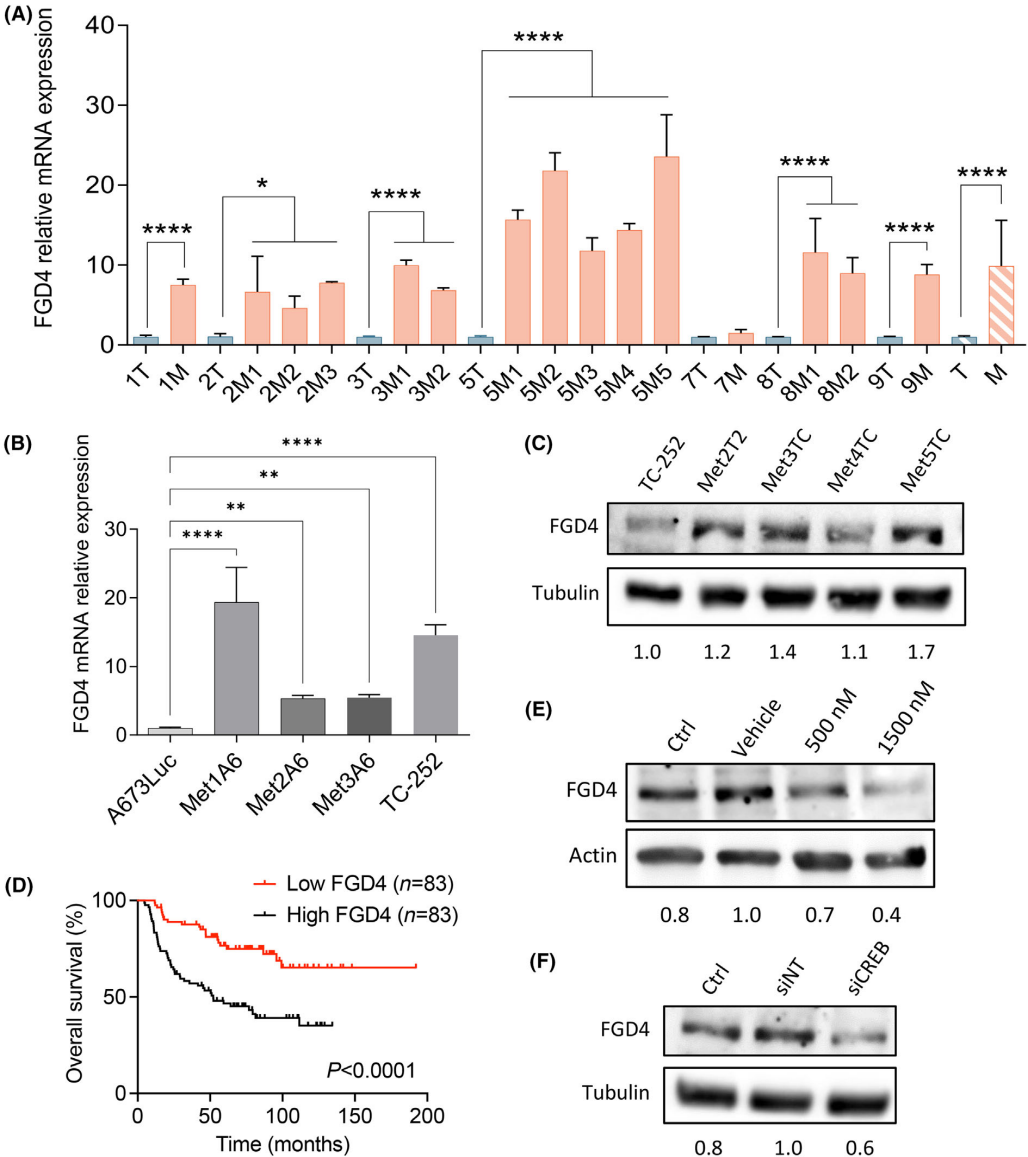
Expression of FGD4 is associated to EWS metastases and survival. (A) qPCR validation of *FGD4* expression on EWS A673 tumors (blue) and metastases (orange) on samples used for the array (1T‐3T, 5T) and extra cohort of EWS A673 samples (7T‐9T). Mean expression values for tumors (T) and metastases (M) plotted (stripped). (B) Expression of *FGD4* by qPCR on metastasis‐derived primary cell lines from *in vivo* lung A673Luc model. qPCR values normalized to housekeeping gene (*PPIA*) and expressed in comparison to paired tumor/cell line expression or A673Luc control. (C) Protein levels of FGD4 evaluated by western blot in four metastasis‐derived primary cell lines from *in vivo* lung TC‐252 metastases. Tubulin as loading control. Numbers indicative of FGD4 ratio normalized to tubulin, respective to TC‐252. (D) Kaplan–Meier curve showing that higher expression of *FGD4* is associated to worse overall survival on EWS patients (*n* = 166). (E, F) Expression of FGD4 is in part regulated by CREB1 signaling, as treatment of TC‐252 cells with CREB1 inhibitor 666‐15 (E) or CREB1 transient silencing (F) results in a reduction of FGD4 protein levels. Tubulin as loading control. Numbers indicative of FGD4 ratio normalized to tubulin, respective to TC‐252 vehicle or siNT control. Three independent replicates were performed for each experiment. ANOVA test. Error bars indicate SEM. Statistical differences as: *P ≤ 0.05, **P ≤ 0.01, ***P ≤ 0.001, ****P ≤ 0.0001. EWS, Ewing sarcoma; siNT, non‐targeting siRNA.

Throughout this multi‐omics study on EWS primary tumor and metastatic samples, *FGD4* appeared both as a top candidate in transcriptomic and methylomic metastatic data. Therefore, we decided to study its involvement in EWS metastatic profile further. Gene expression analyses confirmed that *FGD4* is higher expressed in metastatic samples compared to paired primary tumors of the A673 *in vivo* model (mean tumor 1.0 ± 0.1 vs. mean metastasis 9.9 ± 5.7 *P*‐value <0.0001; Fig. 8A). This was also observed in the metastasis‐derived A673Luc primary cultures (A673Luc 1.0 ± 0.1 vs. Met1A6 19.3 ± 5.1 (*P*‐value <0.0001), vs. Met2A6 5.3 ± 0.5 (*P*‐value = 0.008), vs. Met3A6 5.4 ± 0.4 (*P*‐value = 0.006); Fig. 8B) and the TC‐252 metastatic cell line compared to A673Luc EWS cells (A673Luc 1.0 ± 0.1 vs. TC‐252 14.6 ± 1.5) (*P*‐value <0.0001; Fig. 8B). To rule out the possibility that this increase in *FGD4* expression would be A673 cell line‐dependent, we studied FGD4 protein levels in TC‐252 metastatic‐derived cell cultures (Fig. 8C) and observed an increase in its expression compared to TC‐252 cell line. Moreover, high *FGD4* expression was associated with worse survival in a cohort of 166 EWS patients (*P*‐value <0.0001; Fig. 8D). This was further validated in two different EWS patient cohorts (GSE17679 and GSE63157; Fig. S6A,B). When dividing the cohort by sample type (primary tumors vs. metastases), the metastatic samples presented significantly higher *FGD4* expression (*P*‐value = 0.0252; Fig. S6C), further confirming that high FGD4 expression is a characteristic feature of EWS metastases.

When investigating the methylomic profile of EWS samples, *FGD4* was amongst the top genes that had its promoter hypomethylated in metastatic samples compared to primary tumors (Fig. 3G; Fig. S3B). Interestingly, *in silico* analysis identified a putative association between *FGD4* hypomethylated promoter and CREB1 signaling via *C/EBPβ*. To investigate this link, we studied the expression of *FGD4* in EWS cells treated with 666‐15 CREB1 inhibitor (Fig. 8E) or with transient silencing against *CREB1* (siCREB1; Fig. 8F). As shown, both CREB1 suppressive treatments resulted in a decrease of FGD4 protein expression levels compared to vehicle or siNT control. These results suggest a link between FGD4 expression, the methylation status of its promoter and CREB1 signaling.

## 4 Discussion

In this study we present a multi‐omics approach to investigate the molecular mechanisms behind cancer progression and metastasis in EWS samples. Combining transcriptomics, methylomics and proteomics techniques, we have profiled EWS primary tumors, metastases, and cell lines. We have identified signaling pathways and specific candidate genes that could be important for EWS progression, being validated in different EWS models. Importantly, this is the first time a multi‐omics approach on primary tumors and metastases in EWS has been performed, as previous studies in the EWS field pursuing a similar approach were done on established cell cultures [21, 22].

Our main aim was to understand the changes that occur in EWS cells from the primary tumor to distant metastases. It is well known that cancer cells have to overcome different aspects of the host's biology in order to survive and escape to distant sites (metastasis) [61, 62]. Our focus was solely on the changes occurring within a tumor, and what molecular mechanisms cells exploit to invade to distant organs. For this reason, the samples used in this multi‐omics study were obtained from an EWS spontaneous metastasis mouse model developed in our laboratory, using only one EWS cell line (A673) [23]. However, validation studies were performed in other EWS cell models (TC‐252 *in vivo* model and TC‐252 metastasis‐derived cultures) or other EWS cell lines. Therefore, all possible heterogeneity across samples resulting from different cell lines or patients was removed from the *in silico* analyses. This is fundamental as it means that all changes described here between cells (A673 before injection), primary tumors (A673 masses in the gastrocnemius), and metastases (A673 lung masses after euthanasia) are due to cellular adaptation and interaction with the microenvironment. This is confirmed in the signaling pathways identified in tumors and metastases, as the firsts are associated to cell cycle processes and cytoskeleton modulation, while the latter are linked to immune factors such as IFNα. These results agree with cancer cells being dynamic masses that adapt their phenotype depending on their needs. Cells from the primary tumor could be exploiting signaling cascades associated to cell proliferation, whereas metastatic cells are more into migration and invasion, and playing with the tumor microenvironment to help on the pre‐metastatic niche formation [63]. Literature has generally linked immune modulation via IFNα and TNFα towards tumor suppression in EWS [64, 65]. Nevertheless, a growing body of evidence suggests that the immune system, throughout different cytokines, can modulate the tumor microenvironment and prepare it for disease progression [61, 66, 67, 68]. Cancer cells could be modulating different populations of lymphocytes (B cells or NK cells) or monocytes (macrophages) in order to prepare the pre‐metastatic niche [69]. In addition, the immune system *per se* could be modulating cancer cells into becoming more aggressive, either via pro‐inflammatory signaling cascades or induction of DNA damage [70, 71]. It is important to mention that for the spontaneous metastasis mouse model athymic mice were used, which lack the thymus (no T cells) but still have B and NK cells, thus providing some innate immunity modulation to the tumor microenvironment. Therefore, this putative role of different immune modulators towards a pro‐metastatic phenotype in EWS should be investigated further, as it could shed some light into how EWS cells progress. For this, it would be interesting to use spatial transcriptomics on patient samples, a novel technology that combines the transcriptomic profile with the spatial information of the tissue. Using this, we would fully understand the involvement of the stroma and immune cells towards tumor modulation and disease progression, associated to the clinical profile of EWS patients.

Our study has analyzed EWS samples from three different perspectives: mRNA, protein, and methylation status. Through this approach, large lists of differentially expressed/methylated candidates have been identified. Although identified on an EWS A673 *in vivo* model, results have been validated in other EWS cellular models, including TC‐252 *in vivo* samples and primary cultures. We hope the work presented here will serve the EWS community to understand EWS progression and help their projects move forward. Other omics studies in the past started with big datasets of omics data from EWS samples but ended focusing on specific candidates and signaling pathways that were found to be important in EWS [15, 17, 72, 73]. Throughout our multi‐omics study, we have chosen different genes of interest to validate their putative role in EWS biology, although many more could have fallen into that category.

In our proteomics study we identified the phosphorylation of CREB1 at Ser133 to be enriched in EWS metastatic samples compared to tumors, which was validated *in vitro*. Its increased expression in metastases and association to worse survival has been described in different cancers [59, 74, 75]. Several *in silico* approaches are trying to define which genes CREB1 is targeting, in order to fully understand the potential of this transcription factor and how it is associated to cancer [58, 59]. In our dataset, we have been able to identify many putative CREB1 gene targets amongst our transcriptomic DEG. Although so far is basically an *in silico* approach based on previous data, it narrows down putative signaling cascades that CREB1 could be modulating in EWS. Of interest, *C/EBPβ* is a transcription factor regulated by CREB1 via binding to its CRE site on the promoter [76, 77, 78]. This transcription factor, in turn, regulates the expression of *FGD4* [79, 80], a gene overexpressed in EWS metastases and whose promoter is hypomethylated in our dataset. When evaluating the transcription factors that bind to *FGD4*'s promoter, we detected *C/EBPβ* binding sites nearby the CpG which was differently methylated between EWS tumors and metastases. This would suggest that CREB1 signaling, via *C/EBPβ*, can regulate the expression of *FGD4* in EWS metastases, depending on the methylation status of its promoter. Moreover, clinical datasets in EWS patients have linked *FGD4* expression with worse survival and an increased expression in patient metastases compared to primary tumors, suggesting that indeed FGD4 might be behind aggressiveness of EWS biology. The role of this protein has been associated to cytoskeleton regulation and cell shape, with an association to migration, invasion, tumor progression and survival in cancer [50, 51, 81]. Although the function of this signaling cascade would need to be validated further, it is a putative scenario where the different omics datasets coincide.

Based on our functional validation of CREB1 signaling, we have demonstrated that CREB1 activity is associated to migration and colony forming ability, as the use of the specific 666‐15 CREB1 inhibitor and transient silencing led to a decrease on both features of EWS metastatic cells. Based on the literature, there is an interest in moving CREB1 inhibitors into the clinic, as many malignancies rely on this protein for their aggressive phenotype [82]. However, there are no ongoing clinical trials yet studying how targeting CREB1 or CREB1‐associated targets could help in the treatment of different cancers.

*LOXHD1*, a gene known for its association to hear loss due to a mutation [55], was present amongst the top DEG in EWS metastases. Although there was not much literature linking this gene to cancer nor metastases [83, 84, 85], we decided to investigate its putative role in EWS progression. Interestingly, while investigating *in vitro* its role on the metastatic phenotype of EWS, studies linking LOXHD1 with EWS metastases and its regulation by EWSR1::FLI1 EWS fusion were published [21, 52]. These results validated our methodology, as independent samples and techniques from different groups have resulted in identifying LOXHD1 to be important for EWS metastases. Moreover, we identified that its methylation status is different between tumors and metastases, which suggests a methylomic regulation of its expression. All this evidence strongly suggests that LOXHD1 might be an important regulator for EWS disease progression.

Multi‐omics studies have the characteristic of generating large amounts of data, and often is hard to focus only on some candidates. However, we have identified several genes overexpressed or hypomethylated in our EWS metastatic samples that have been reported previously in EWS literature. For instance, *ERBB4*, identified in our transcriptomic and proteomic datasets, has been linked to EWS metastasis [86, 87]. The histone demethylase *KDM3A*, amongst the transcripts enriched in EWS metastases, has been associated to migration and metastasis in EWS cells [88, 89, 90, 91]. Other metastasis‐associated genes identified in this multi‐omics approach previously described in the EWS literature are *ABCA6* [91], *IL1RAP* [16], *MEIS1* [19], *MERTK* [92], *NKX2‐2* [93, 94], *SLIT2* [95], and *XG* [96]. Through the different *in silico* functional analysis, we used the IPA software (Qiagen) to investigate our transcriptomic EWS profile and identify pathways and mechanisms that could be regulating EWS, based on previously curated datasets. Interestingly, the *PPP1R1B* gene was identified as the top master regulator of our EWS tumors and metastases distinct profile. This gene, also known as *DARPP‐32*, encodes for a protein phosphatase that has been associated to PKA and PP1 inhibition, and to tumor progression and lower survival rates in different cancers [97, 98, 99, 100, 101, 102]. Moreover, *PPP1R1B* is a cAMP‐regulated phosphoprotein, and literature links its function to CREB1 signaling, affecting its downstream binding to CRE sites and activation of target genes [103, 104]. Therefore, it might be that *PPP1R1B* is important for EWS molecular machinery and is linked to downstream signaling pathways identified in this study.

## 5 Conclusions

In conclusion, this multi‐omics approach has been used to investigate EWS primary tumors and metastases, identifying different pathways that EWS cells could be exploiting for disease progression. Although many data were generated, a range of *in vitro* experiments were included in order to validate the methodology and shed some light into what these results might mean in a biological setting. Our spontaneous metastasis mouse model has proven again to be a good model to study EWS [40, 41], since data obtained on different EWS cell lines agree with what other groups are seeing [16, 19, 21, 52, 86, 87, 88, 89, 90, 91, 92, 93, 94, 95, 96]. Based on the results presented here, further studies are needed to fully decipher the identified candidates (i.e., CREB1, LOXHD1, or FGD4) and find others that could be behind EWS progression. Moreover, a focus on understanding how immune modulation (via IFNα or TNFα) might be helping EWS cells towards metastasis formation is important, as we could be in front of a microenvironment modulation responsible for changing tumors' phenotypes into metastatic‐associated. Altogether, we hope our results help the EWS research community into better understanding the process of EWS tumorigenesis and metastasis, leading to better treatment strategies and improved survival rates for EWS patients.

## Conflict of interest

The authors declare no competing interests.

## Author contributions

Conceptualization, OMT and MCB; methodology, MCB, NCF, and RLA; investigation and interpretation, MCB, SSS, MRL, NCF, JBV, JIB, SML, FCA, TGPG, ADL, RLA, and OMT; writing original draft preparation, MCB; writing review and editing, MCB, SML, ADL, RLA, and OMT; visualization, MCB and NCF; funding acquisition, TGPG, FCA, ADL, and OMT; software, MCB and NCF.

## Supporting information


**Fig. S1.** Principal component analysis (PCA) of EWS samples in our transcriptomic dataset. Distribution of primary tumors (green), metastases (purple) and cell lines (pale orange) based on PCA1‐2. Some metastases are closer to primary tumors (i.e. 4‐5M) compared to others (i.e. 1‐2‐3M), indicating heterogeneity amongst EWS metastatic samples. EWS, Ewing sarcoma.

**Fig. S2.** Pathway enrichment identified in our transcriptomic EWS dataset. (A) Graphical summary on the pathways enriched on our transcriptomic dataset identified using the Ingenuity Pathway Analysis (IPA). (B) Example of a master regulator (*PP1R1B*) of our dataset, regulating different signaling cascades present in the transcriptomics results. Colors indicate predicted activation (orange) and inhibition (blue); observed (from our transcriptomics) increase (red) and decrease (green). EWS, Ewing sarcoma.

**Fig. S3.** Pyrosequencing validation of differentially methylated genes. Pyrosequencing validation of (A) *LOXHD1* and (B) *FGD4* methylation on A673 primary tumors and metastases used on the Infinium MethylationEPIC array. For *LOXHD1* (A), data on TC‐252 primary tumors and metastases is also included. Results on genome track, indicating location of differential methylation and information on methylation levels for each sample. T as tumor, M1‐3 as metastases.

**Fig. S4.** Decitabine treatment confirms a methylomic regulation of LOXHD1. (A) Pyrosequencing analysis of A673 and TC‐252 cells treatment with Decitabine (72 h) compared to vehicle control confirms a reduction of methylation levels on *LOXHD1* promoter on both cell lines. (B) mRNA expression levels (qPCR) of *LOXHD1* in A673 cells after treatment with Decitabine (72 h) confirm a regulation of *LOXHD1* via methylation. qPCR values normalized to housekeeping gene (*PPIA*) and expressed in comparison to vehicle control. Mean of 3 independent repeats.

**Fig. S5.** CREB1 inhibitor treatment effects on EWS cells. (A) Expression of CREB1 and phospho‐CREB1 (Ser133) after treatment with CREB1 inhibitor 666‐15 in Met1A6 primary culture (top) and TC‐252 metastatic cell line (bottom). Tubulin as loading control. Western blot image representative of 3 independent repeats. 48 h of treatment. Expression of phospho‐CREB1 normalized to tubulin, respective to vehicle control. (B) Transient silencing of *CREB1* on TC‐252 cells after 48 h evaluated by Western blot. Tubulin as loading control. Expression of phospho‐CREB1 normalized to tubulin, respective to siNT. (C) Migration ability of TC‐252 EWS cells is decreased when CREB1 is silenced (transient silencing) after 72 h of silencing. Results based on migrated cells after 48 h on Boyden chamber assay. Pictures of the bottom of chambers and their quantification (imagej). (D) Proliferation assay on Met1A6 and TC‐252 cells treated with a range of concentrations of CREB1 inhibitor 666‐15 (10–1500 nm). Values from 3 independent repeats. Data normalized to 24 h vehicle treatment. Statistics indicative of differences on proliferation compared to vehicle control of each time point calculated using ANOVA. Statistical differences as: *P ≤ 0.05, **P ≤ 0.01, ***P ≤ 0.001, ****P ≤ 0.0001. EWS, Ewing sarcoma; siNT, non‐targeting siRNA.

**Fig. S6.** Clinical validation of FGD4 expression on EWS samples. Kaplan‐Meier curves of overall survival in the (A) GSE17679 and (B) GSE63157 EWS publicly available datasets stratified based on *FGD4* expression values (high = red, low = blue). Results from R2 online software (https://hgserver1.amc.nl). (C) Gene expression (FGD4) according to sample origin (primary tumor, metastasis) on a cohort of 166 EWS patients. EWS, Ewing sarcoma.

**Table S1.** Raw proteomics data on 1T‐1M sample comparison. Cells injected in mice (*Ax*), primary tumors obtained from the spontaneous metastasis mouse model (*xT*) and lung metastases (*xM*) were obtained from different mice. Samples were analyzed using transcriptomics (Clariom^TM^ D), methylomics (Infinium MethylationEPIC) and proteomics (Phospoho Explorer) arrays, as indicated by “X”. Some samples (both A673 and TC‐252 origin) were only used for validation of candidate genes (extra cohort of samples). Number indicative of same mice (i.e. 1M is metastasis from 1T tumor). Primary tumor that failed quality control and was not used for analysis is indicated as F (fail). EWS, Ewing sarcoma.

**Table S2.** Raw proteomics data on 2T‐2M sample comparison. A comprehensive list of all proteins and phospho‐proteins included in the proteomics phosphoarray is provided. Average signal intensity and normalized values are shown. Fold‐change between metastasis and tumor sample was used for the analyses.

**Table S3.** Raw proteomics data on 3T‐3M sample comparison. A comprehensive list of all proteins and phospho‐proteins included in the proteomics phosphoarray is provided. Average signal intensity and normalized values are shown. Fold‐change between metastasis and tumor sample was used for the analyses.

**Table S4.** Raw proteomics data on all T‐ all M samples comparison. A comprehensive list of all proteins and phospho‐proteins included in the proteomics phosphoarray is provided. Average signal intensity and normalized values are shown. Fold‐change between metastasis and tumor sample was used for the analyses.

**Table S5.** Detailed description of EWS samples used. A comprehensive list of all proteins and phospho‐proteins included in the proteomics phosphoarray is provided. Average signal intensity and normalized values are shown. Fold‐change between metastasis and tumor sample was used for the analyses.

## Data Availability

Additional data are presented as supplementary Figures. The datasets supporting the conclusions of this article are available in the GEO repository (methylomics: GSE245203; transcriptomics: GSE245203; proteomics: supplied as Tables [Link], [Link], [Link], [Link]).
