# Supplementary figures and images for: Multi‐omics profiling reveals key factors involved in Ewing sarcoma metastasis
Source: Mol Oncol. 2025 Jan 5;19(4):1002–28. doi: 10.1002/1878-0261.13788 (PMC11977646; doi:10.1002/1878-0261.13788)

Principal component analysis (PCA)

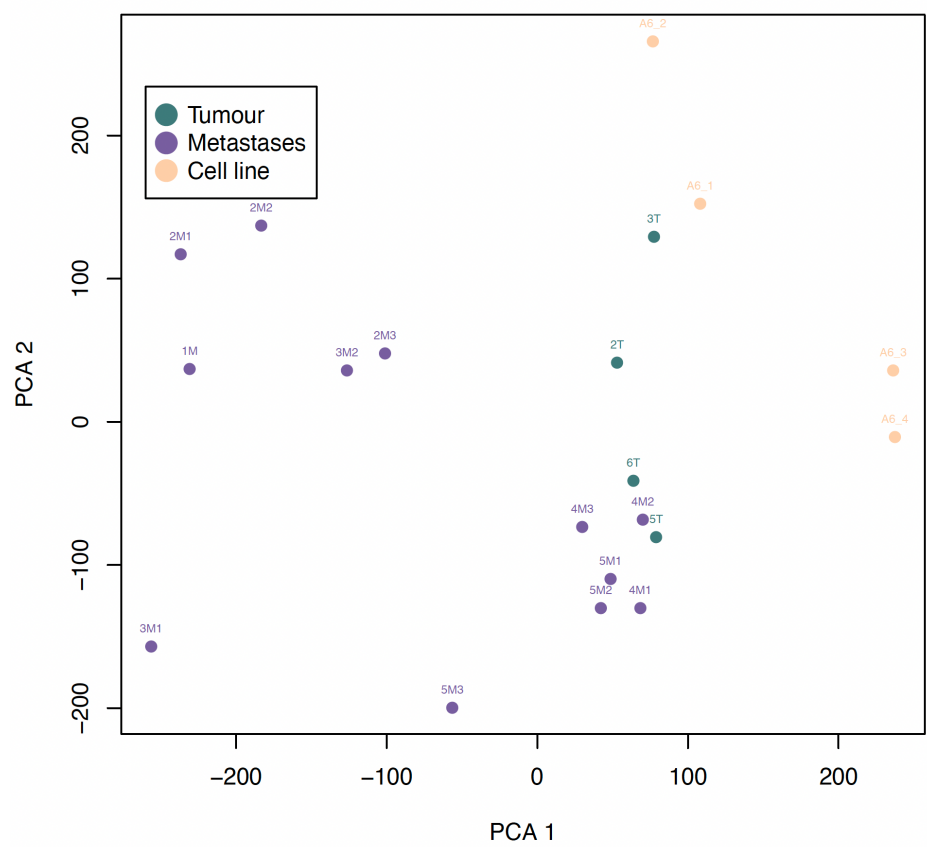

Supplement: Supplementary file 1 — Fig. S1. Principal component analysis (PCA) of EWS samples in our transcriptomic dataset. Distribution of primary tumors (green), metastases (purple) and cell lines (pale orange) based on PCA1‐2. Some metastases are closer to primary tumors (i.e. 4‐5M) compared to others (i.e. 1‐2‐3M), indicating heterogeneity amongst EWS metastatic samples. EWS, Ewing sarcoma. [file MOL2-19-1002-s010.pdf]

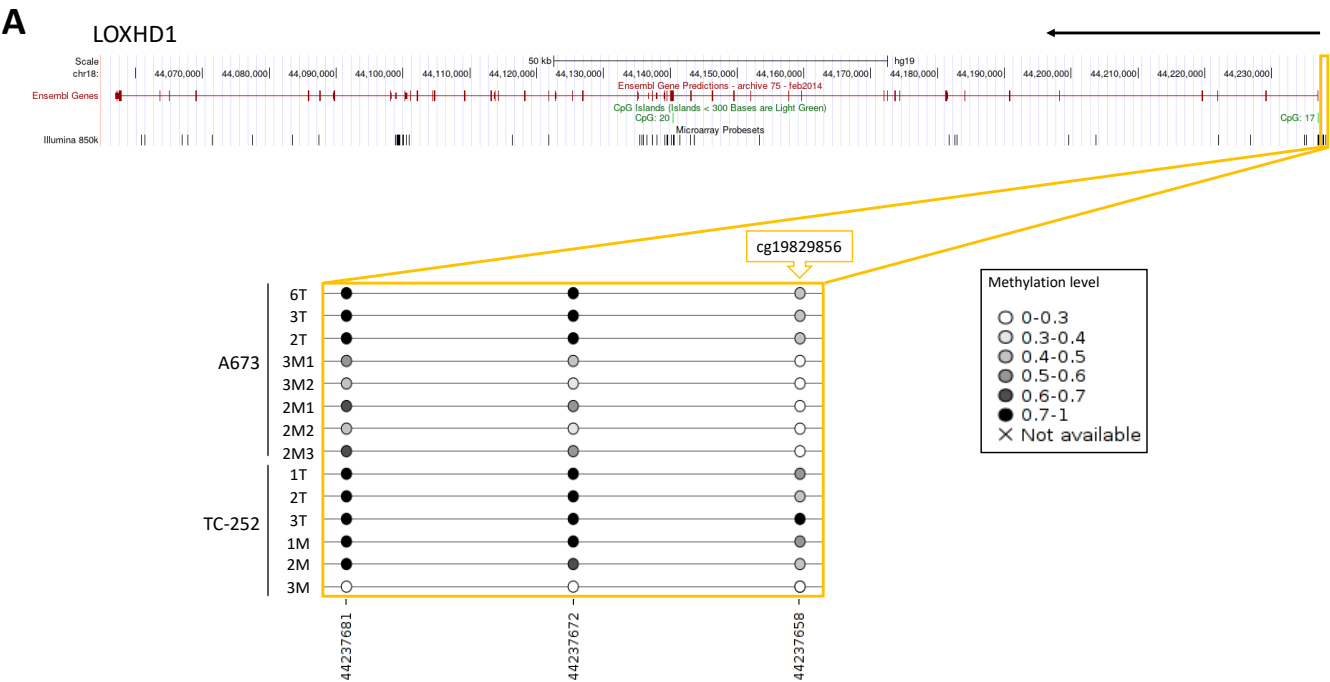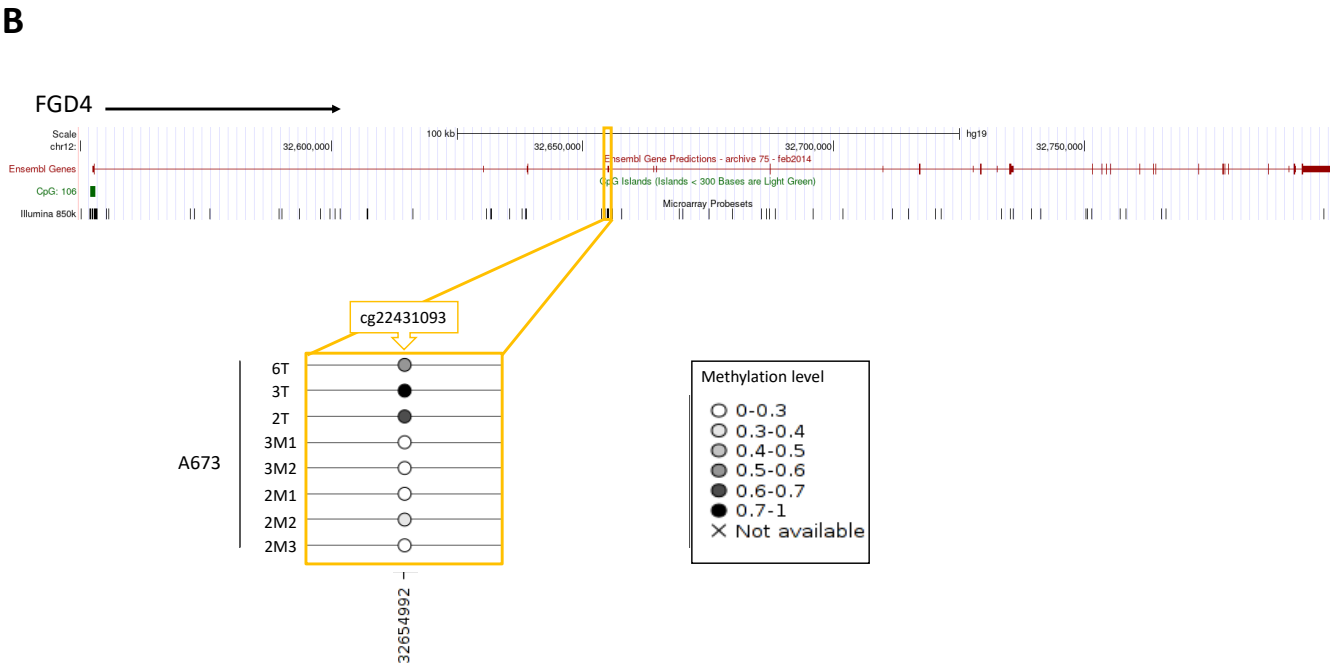

Supplement: Supplementary file 3 — Fig. S3. Pyrosequencing validation of differentially methylated genes. Pyrosequencing validation of (A) LOXHD1 and (B) FGD4 methylation on A673 primary tumors and metastases used on the Infinium MethylationEPIC array. For LOXHD1 (A), data on TC‐252 primary tumors and metastases is also included. Results on genome track, indicating location of differential methylation and information on methylation levels for each sample. T as tumor, M1‐3 as metastases. [file MOL2-19-1002-s007.pdf]

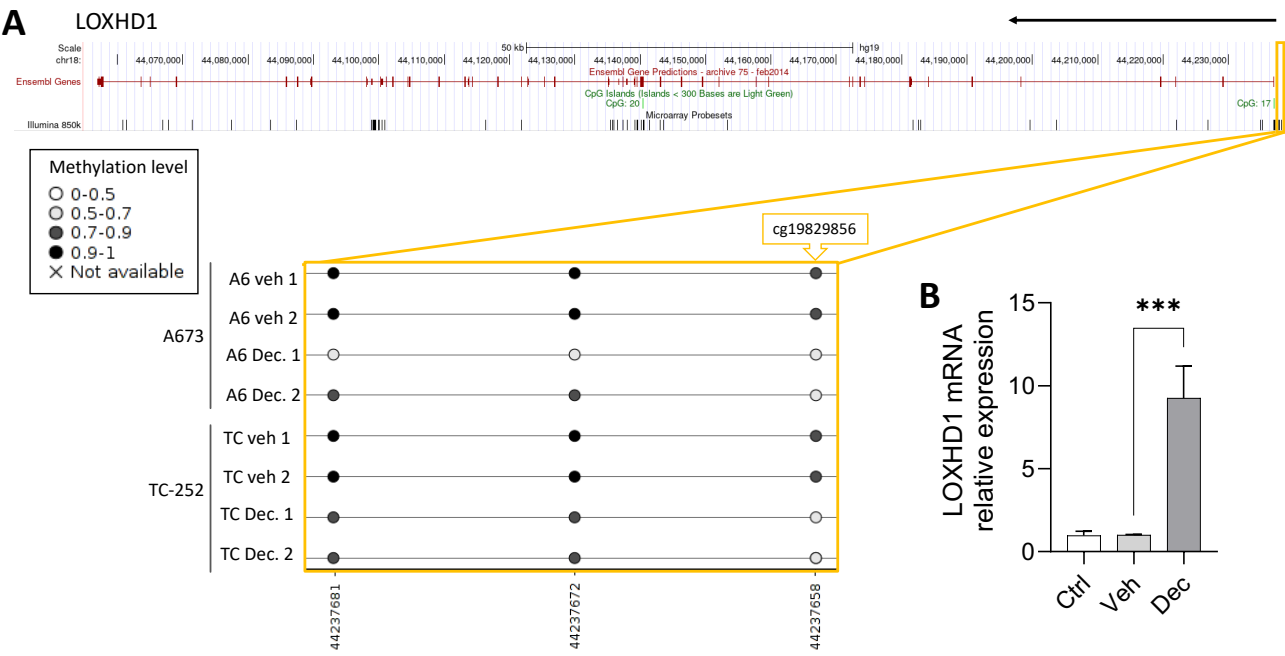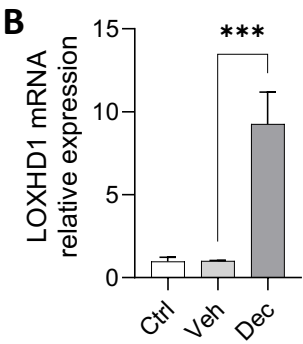

Supplement: Supplementary file 4 — Fig. S4. Decitabine treatment confirms a methylomic regulation of LOXHD1. (A) Pyrosequencing analysis of A673 and TC‐252 cells treatment with Decitabine (72 h) compared to vehicle control confirms a reduction of methylation levels on LOXHD1 promoter on both cell lines. (B) mRNA expression levels (qPCR) of LOXHD1 in A673 cells after treatment with Decitabine (72 h) confirm a regulation of LOXHD1 via methylation. qPCR values normalized to housekeeping gene (PPIA) and expressed in comparison to vehicle control. Mean of 3 independent repeats. [file MOL2-19-1002-s008.pdf]

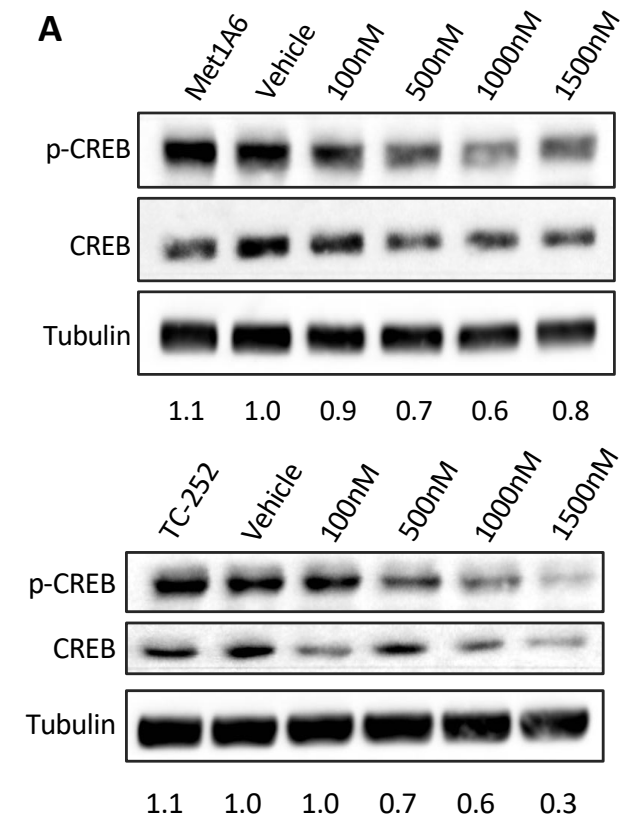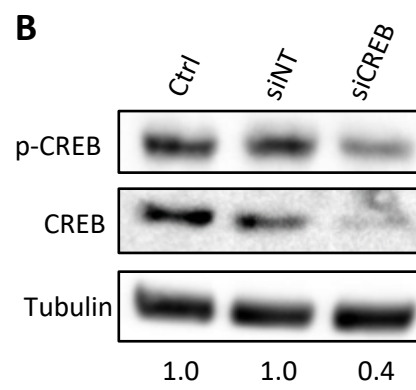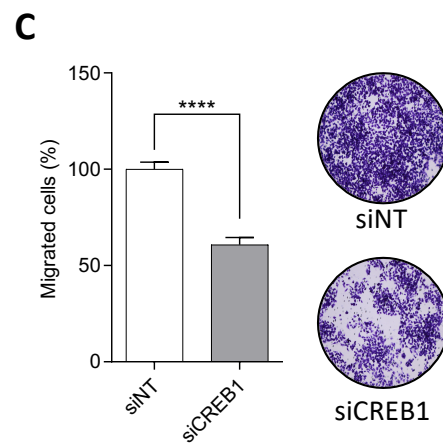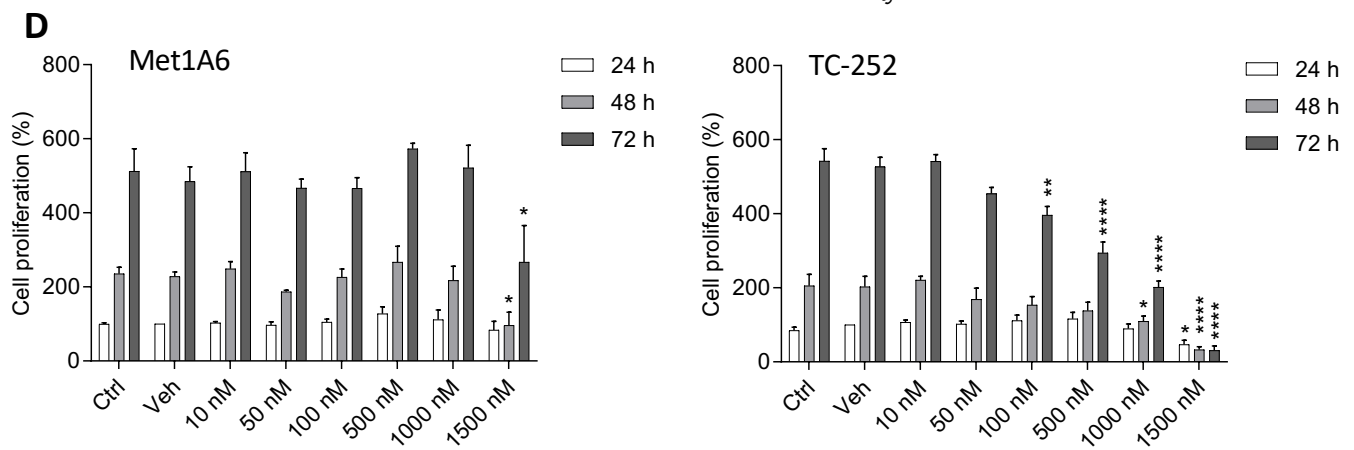

Supplement: Supplementary file 5 — Fig. S5. CREB1 inhibitor treatment effects on EWS cells. (A) Expression of CREB1 and phospho‐CREB1 (Ser133) after treatment with CREB1 inhibitor 666‐15 in Met1A6 primary culture (top) and TC‐252 metastatic cell line (bottom). Tubulin as loading control. Western blot image representative of 3 independent repeats. 48 h of treatment. Expression of phospho‐CREB1 normalized to tubulin, respective to vehicle control. (B) Transient silencing of CREB1 on TC‐252 cells after 48 h evaluated by Western blot. Tubulin as loading control. Expression of phospho‐CREB1 normalized to tubulin, respective to siNT. (C) Migration ability of TC‐252 EWS cells is decreased when CREB1 is silenced (transient silencing) after 72 h of silencing. Results based on migrated cells after 48 h on Boyden chamber assay. Pictures of the bottom of chambers and their quantification (imagej). (D) Proliferation assay on Met1A6 and TC‐252 cells treated with a range of concentrations of CREB1 inhibitor 666‐15 (10–1500 nm). Values from 3 independent repeats. Data normalized to 24 h vehicle treatment. Statistics indicative of differences on proliferation compared to vehicle control of each time point calculated using ANOVA. Statistical differences as: *P ≤ 0.05, **P ≤ 0.01, ***P ≤ 0.001, ****P ≤ 0.0001. EWS, Ewing sarcoma; siNT, non‐targeting siRNA. [file MOL2-19-1002-s004.pdf]

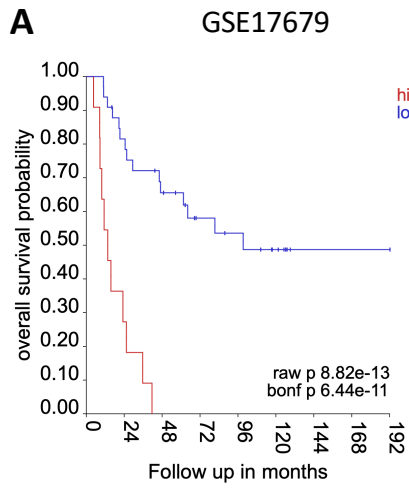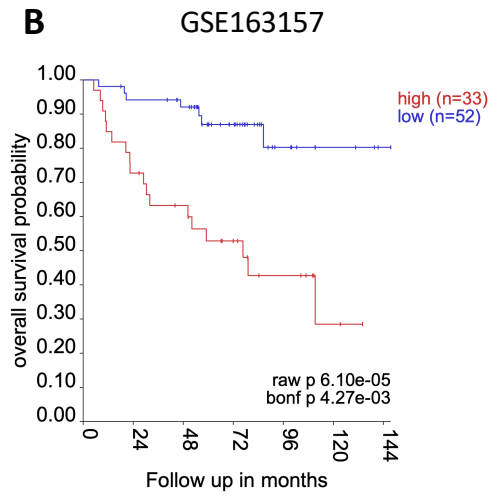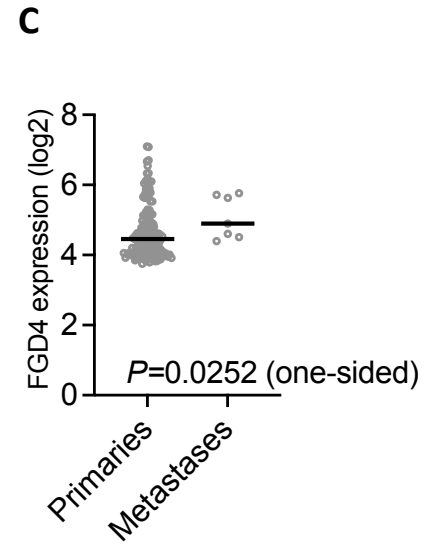

Supplement: Supplementary file 6 — Fig. S6. Clinical validation of FGD4 expression on EWS samples. Kaplan‐Meier curves of overall survival in the (A) GSE17679 and (B) GSE63157 EWS publicly available datasets stratified based on FGD4 expression values (high = red, low = blue). Results from R2 online software (https://hgserver1.amc.nl). (C) Gene expression (FGD4) according to sample origin (primary tumor, metastasis) on a cohort of 166 EWS patients. EWS, Ewing sarcoma. [file MOL2-19-1002-s002.pdf]
