# Supplementary material for: Multi‐omics profiling reveals key factors involved in Ewing sarcoma metastasis
Source: Mol Oncol. 2025 Jan 5;19(4):1002–28. doi: 10.1002/1878-0261.13788 (PMC11977646; doi:10.1002/1878-0261.13788)
Supplement: Supplementary file 2 — Fig. S2. Pathway enrichment identified in our transcriptomic EWS dataset. (A) Graphical summary on the pathways enriched on our transcriptomic dataset identified using the Ingenuity Pathway Analysis (IPA). (B) Example of a master regulator (PP1R1B) of our dataset, regulating different signaling cascades present in the transcriptomics results. Colors indicate predicted activation (orange) and inhibition (blue); observed (from our transcriptomics) increase (red) and decrease (green). EWS, Ewing sarcoma. [file MOL2-19-1002-s011.pdf]

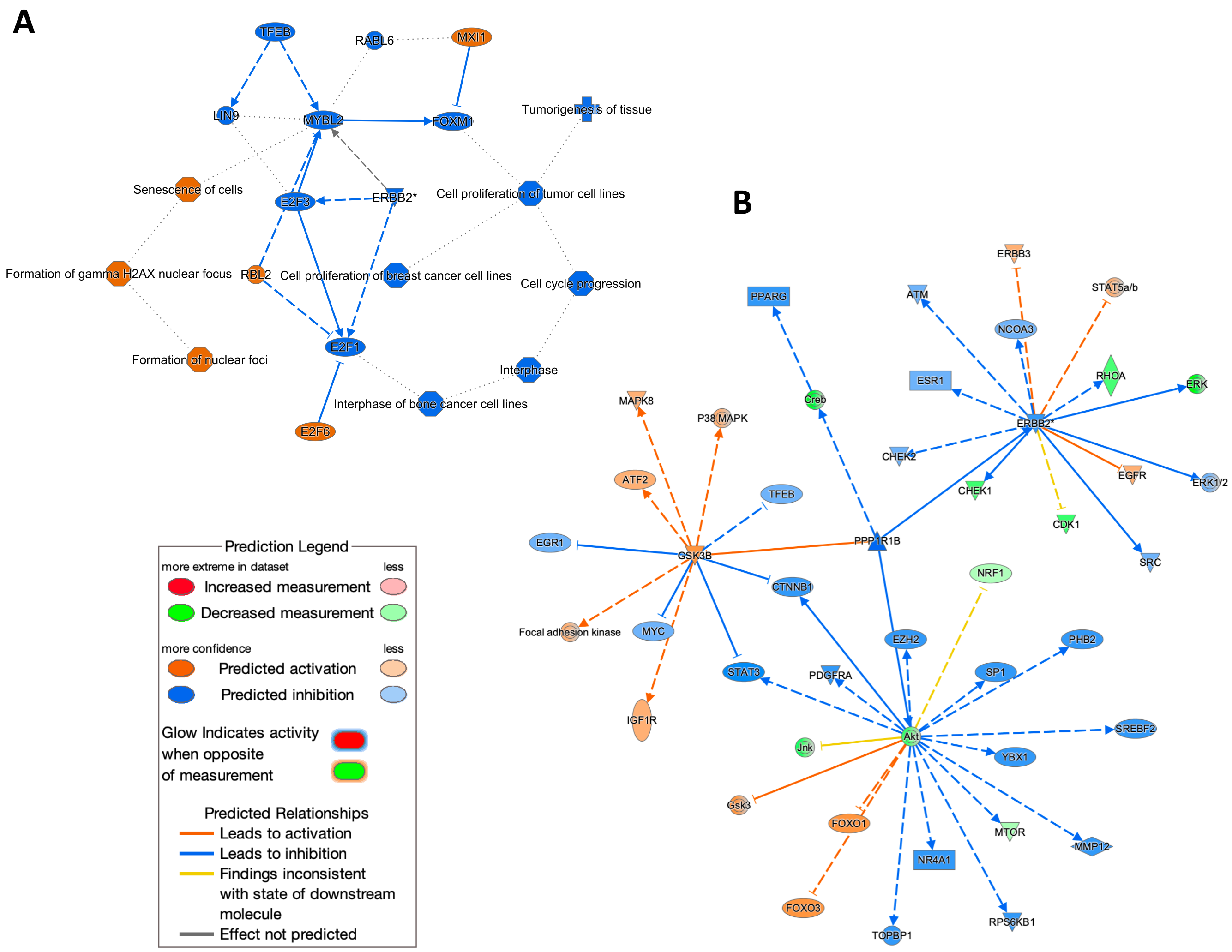
A
TFEB
RABL6
MXI1
LIN9
MYBL2
FOXM1
Tumorigenesis of tissue
Senescence of cells
E2F3
ERBB2*
Cell proliferation of tumor cell lines
Formation of gamma H2AX nuclear focus
RBL2
Cell proliferation of breast cancer cell lines
Cell cycle progression
E2F1
Formation of nuclear foci
Interphase
Interphase of bone cancer cell lines
E2F6
B
ERBB3
PPARG
ATM
STAT5a/b
NCOA3
ESR1
RHOA
ERK
Creb
MAPK8
P38 MAPK
ERBB2*
CHEK2
ATF2
CHEK1
EGFR
ERK1/2
TFEB
CDK1
EGR1
GSK3B
PPP1R1B
SRC
NRF1
CTNNB1
Focal adhesion kinase
MYC
EZH2
PHB2
STAT3
PDGFRA
SP1
IGF1R
SREBF2
Jnk
Akt
YBX1
Gsk3
FOXO1
MTOR
MMP12
NR4A1
FOXO3
RPS6KB1
TOPBP1
Prediction Legend
more extreme in dataset
less
Increased measurement
Decreased measurement
more confidence
less
Predicted activation
Predicted inhibition
Glow Indicates activity when opposite of measurement
Predicted Relationships
Leads to activation
Leads to inhibition
Findings inconsistent with state of downstream molecule
Effect not predicted
